# Phylogenomics reveals subfamilies of fungal nonribosomal peptide synthetases and their evolutionary relationships

**DOI:** 10.1186/1471-2148-10-26

**Published:** 2010-01-26

**Authors:** Kathryn E Bushley, B Gillian Turgeon

**Affiliations:** 1 Department of Plant Pathology & Plant-Microbe Biology, 334 Plant Science Bldg. Cornell University, Ithaca, NY, 14853, USA

## Abstract

**Background:**

Nonribosomal peptide synthetases (NRPSs) are multimodular enzymes, found in fungi and bacteria, which biosynthesize peptides without the aid of ribosomes. Although their metabolite products have been the subject of intense investigation due to their life-saving roles as medicinals and injurious roles as mycotoxins and virulence factors, little is known of the phylogenetic relationships of the corresponding NRPSs or whether they can be ranked into subgroups of common function. We identified genes (*NPS*) encoding NRPS and NRPS-like proteins in 38 fungal genomes and undertook phylogenomic analyses in order to identify fungal NRPS subfamilies, assess taxonomic distribution, evaluate levels of conservation across subfamilies, and address mechanisms of evolution of multimodular NRPSs. We also characterized relationships of fungal NRPSs, a representative sampling of bacterial NRPSs, and related adenylating enzymes, including α-aminoadipate reductases (AARs) involved in lysine biosynthesis in fungi.

**Results:**

Phylogenomic analysis identified nine major subfamilies of fungal NRPSs which fell into two main groups: one corresponds to *NPS *genes encoding primarily mono/bi-modular enzymes which grouped with bacterial NRPSs and the other includes genes encoding primarily multimodular and exclusively fungal NRPSs. AARs shared a closer phylogenetic relationship to NRPSs than to other acyl-adenylating enzymes. Phylogenetic analyses and taxonomic distribution suggest that several mono/bi-modular subfamilies arose either prior to, or early in, the evolution of fungi, while two multimodular groups appear restricted to and expanded in fungi. The older mono/bi-modular subfamilies show conserved domain architectures suggestive of functional conservation, while multimodular NRPSs, particularly those unique to euascomycetes, show a diversity of architectures and of genetic mechanisms generating this diversity.

**Conclusions:**

This work is the first to characterize subfamilies of fungal NRPSs. Our analyses suggest that mono/bi-modular NRPSs have more ancient origins and more conserved domain architectures than most multimodular NRPSs. It also demonstrates that the α-aminoadipate reductases involved in lysine biosynthesis in fungi are closely related to mono/bi-modular NRPSs. Several groups of mono/bi-modular NRPS metabolites are predicted to play more pivotal roles in cellular metabolism than products of multimodular NRPSs. In contrast, multimodular subfamilies of NRPSs are of more recent origin, are restricted to fungi, show less stable domain architectures, and biosynthesize metabolites which perform more niche-specific functions than mono/bi-modular NRPS products. The euascomycete-only NRPS subfamily, in particular, shows evidence for extensive gain and loss of domains suggestive of the contribution of domain duplication and loss in responding to niche-specific pressures.

## Background

Nonribosomal peptide synthetases (NRPSs) are multimodular megasynthases which catalyze biosynthesis of small bioactive peptides (NRPs) via a thiotemplate mechanism independent of ribosomes [1-5]. NRPS encoding genes (*NPS*s) are plentiful in fungi and bacteria but are not known in plants or animals. The enzymes they encode biosynthesize a staggering diversity of chemical products because their substrates can include both D and L forms of the 20 amino acids used in ribosomal protein synthesis, as well as non-proteinogenic amino acids such as ornithine, imino acids, and hydroxy acids such as α-aminoadipic and α-butyric acids [1]. The natural functions of most NRPs for producing organisms are largely unknown, although recently it has become clearer that they play fundamental roles in fungal reproductive and pathogenic development, morphology, cell surface properties, stress management, and nutrient procurement [6-15] in addition to better-known roles as toxins/mycotoxins involved in plant or animal pathogenesis or as life-saving pharmaceuticals such as antibiotics, immunosuppressants, and anticancer agents.

NRPSs use a set of core domains, known as a module, to accomplish peptide synthesis. A minimal module consists of three core domains: an adenylation (A) domain which recognizes and activates the substrate via adenylation with ATP, a thiolation (T) or peptidyl carrier protein (PCP) domain which binds the activated substrate to a 4'-phosphopantetheine (PP) cofactor via a thioester bond and transfers the substrate to a condensation (C) domain which catalyzes peptide bond formation between adjacent substrates on the megasynthase complex [1]. Several specialized C-terminal domains involved in chain termination and release of the final peptide product have also been identified [16,17]. In bacteria, chain release is most commonly effected by a thioesterase (TE) domain [18], which releases the peptide by either hydrolysis or internal cyclization [16,17,19]. In fungi, only a few NRPSs, such as the ACV synthetases, are known to release products via a TE domain and chain release is carried out by a variety of mechanisms, two of which predominate and occur less frequently in bacterial systems: 1) a terminal C domain, which catalyzes release by inter- or intra-molecular amide bond formation [16], and 2) a thioesterase NADP(H) dependent reductase (R) domain [20-23], which catalyzes reduction with NADPH to form an aldehyde. An additional mechanism, which has been reported only in biosynthesis of fungal ergot alkaloids, involves nonenzymatic cyclization by formation of a diketopiperazine ring [16,24].

NRPSs may contain additional modifying domains which alter the substrate during NRPS biosynthesis: 1) an epimerization (E) domain which catalyzes epimerization of an amino acid from the L to the D configuration [25], 2) an N-methylation (M) domain (methyltransferase) which catalyzes transfer of a methyl group from an S-adenosylmethionine to an α-amino of the amino acid substrate, and 3) a specialized C domain termed a cyclization (Cyc) domain which catalyzes formation of oxazoline or thiazoline rings by internal cyclization of cysteine, serine, or threonine residues [26]. Additional tailoring enzymes which are not part of the NRPS may modify either the substrate or the final peptide product by glycosylation, hydroxylation, acylation, or halogenation [27,28].

NRPSs may be monomodular, consisting of a single A-T-C module, or multimodular, consisting of repeated A-T-C modules. The suite of 14 NRPSs found in the genome of the Dothideomycete *Cochliobolus heterostrophus *is representative of the diversity of *NPS *genes in filamentous ascomycetes in that it contains a representative from most currently recognized groups of fungal NRPSs [10,6], and, with the exception of duplicated copies of *ChNPS12*, the modular domain architectures of each encoded enzyme are distinct (Additional file 1). In addition to mono- and multi-modular *NPSs*, a hybrid gene (*ChNPS7*; *PKS24*) encoding an incomplete NRPS module (A-T) fused to a polyketide synthase (PKS) unit is present [10,29]. Hybrid PKS;NRPS synthetases (e.g. ACE1, SYN2 in *Magnaporthe oryzae*), the reverse organization of ChNPS7;PKS24, are more common in filamentous fungi as well as in bacteria [30-34], although *C. heterostrophus *lacks a representative. PKSs, found in fungi, bacteria, and plants are large megasynthases, related to fatty acid synthases, that biosynthesize small molecule polyketides with as diverse natural functions as NRPS metabolites.

The evolutionary mechanisms giving rise to genes encoding enzymes with such diverse modular architectures are clearly complex. Likely mechanisms include: 1) tandem duplication and loss of individual modules or domains, 2) gene fusion/fission, and 3) recombination and/or gene conversion of individual modules or domains either within the same *NPS *or between different *NPS*s. It has been suggested that genes involved in secondary metabolite (small molecule) biosynthesis tend to be located in subtelomeric regions, a factor which may contribute to their rapid evolution by the aforementioned mechanisms [35,36].

*NPS*s are generally recognized as a rapidly evolving gene class in fungi leading to few clearly identifiable orthologs between species and highly discontinuous distributions [10,37,38]. However, as has been observed for members of other eukaryotic gene families (e.g., major histocompatibility complex [39], immune response [40], zinc-finger [41], reproductive [42], olfactory/chemosensory [43-47], MADS-box [48], and F-box gene families [49] among others), within each family, conservation and rates of gene duplication and loss are likely to vary among subgroups of genes encoding proteins of different function. In fact, some *C. heterostrophus NPSs *(*NPS2*, *NPS4*, *NPS6 *and *NPS10*), are conserved or moderately conserved across euascomycote fungi [8,10,50] and their NRP products are involved in basic cellular functions such as growth and development, reproduction, and pathogenesis [6-8]. The majority of *NPS*s, however, are highly discontinuously distributed across fungal taxa and even closely related species may share only a few homologs. Some, e.g., *Cochliobolus carbonum HTS1*, the gene encoding the NRPS for biosynthesis of HC-toxin [51], and *Alternaria alternata *apple pathotype *AMT*, the gene encoding the NRPS for biosynthesis of AM-toxin [52], appear unique even to one race or pathotype within a single species. These lineage-specific synthetases tend to have more specialized, niche-specific functions.

Higher rates of gene duplication and loss may reflect an adaptive response to selective pressure from pathogens, interactions with other organisms, or other environmental pressures. Recent work suggests that, in fungi, genes involved in responses to stress are more likely to undergo duplication and loss than growth related genes [53]. Thus, we hypothesize that NRPSs with conserved functions involved in growth and development will show less variation in gene copy number and maintain a relatively conserved domain architecture in comparison with NRPSs with more niche-specific functions.

The multimodular structure of NRPSs and the complex mechanisms by which they evolve present challenges to phylogenetic analysis and consequently little work has been done to characterize phylogenetic relationships across this large class of megasynthases or to ask whether subclasses of common function can be identified, based on close relationships with NRPSs whose chemical products are known. In this study, we undertook phylogenomic analyses on a comprehensive dataset of fungal NRPS proteins to: 1) identify major subfamilies, 2) analyze patterns of distribution of these major subfamilies across fungal taxonomic groups, 3) understand relationships among selected bacterial NRPSs, fungal monomodular NRPS/NRPS-like proteins, fungal multimodular NRPSs, and related adenylating enzymes, including α-aminoadipate reductases involved in lysine biosynthesis in fungi, 4) consider mechanisms of evolution of multimodular NRPSs, and 5) analyze patterns of NRPS gene and A domain duplication and loss across fungi.

## Results and Discussion

### Identification and domain structure of candidate NRPSs

Candidate NRPSs extracted from each sequenced genome are listed in Additional file 2. Genus and species abbreviations for all organisms mentioned in this study are shown in Additional file 3. The proposed domain structure for each NRPS, based on searches with our fungal-specific HMMER models (Additional file 4) and the PFAM and Interpro databases, is shown in Additional file 2. The majority of multimodular NRPSs were composed of one or more standard NRPS modules (A-T-C) with or without modifying domains (E, M, etc), while most monomodular NRPSs lacked complete A-T-C modules and consisted of a single A domain or an A-T unit followed by a variety of C-terminal domains, several of which have not previously been identified as core NRPS domains (Additional file 2).

### Phylogenomic analysis and subfamily identification

**Figure 1:**
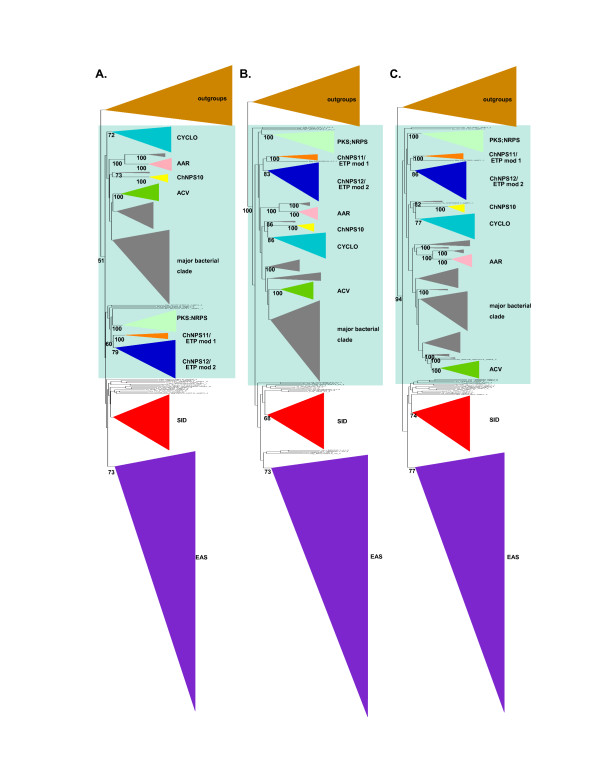
**Cartoons of tree topologies showing major NRPS subfamilies**. All trees reflect phylogenetic analyses of the complete A domain dataset. A. NJ tree using a ML distance matrix created using the WAG plus gamma model. B. ML tree (PhyML) using the WAG plus gamma model. C. ML tree (RAxML) using the RTREVF plus gamma model. Bootstrap support greater than 50% is shown under branches. The light blue rectangle indicates primarily mono/bi-modular NRPS; the SID and EAS subclasses are primarily multimodular. Color coding for subfamilies: brown: adenylating enzyme outgroups; light green: fungal PKS;NRPS hybrid synthetases (PKS;NRPS); dark orange: ChNPS11/ETP module 1 synthetases (ChNPS11/ETP mod 1); dark blue: ChNPS12/ETP module 2 synthetases (ChNPS12/ETP mod 2); yellow: ChNPS10-like synthetases (ChNPS10); light blue: Cyclosporin synthetases (CYCLO); pink: α-aminoadipate reductases (AAR); dark green: ACV synthetases (ACV); red: siderophore synthetases (SID); purple: Euascomycete clade synthetases (EAS). The majority of bacterial sequences (dark gray) group together although they contain a few fungal A domains (ACV synthetases and the NRPS;PKS hybrid (ChNPS7;PKS24) suspected of being horizontally transmitted from bacteria to fungi. The remaining bacterial A domains group with the mono/bi-modular AAR and ChNPS12/ETP mod 2 subfamilies.

**Figure 2:**
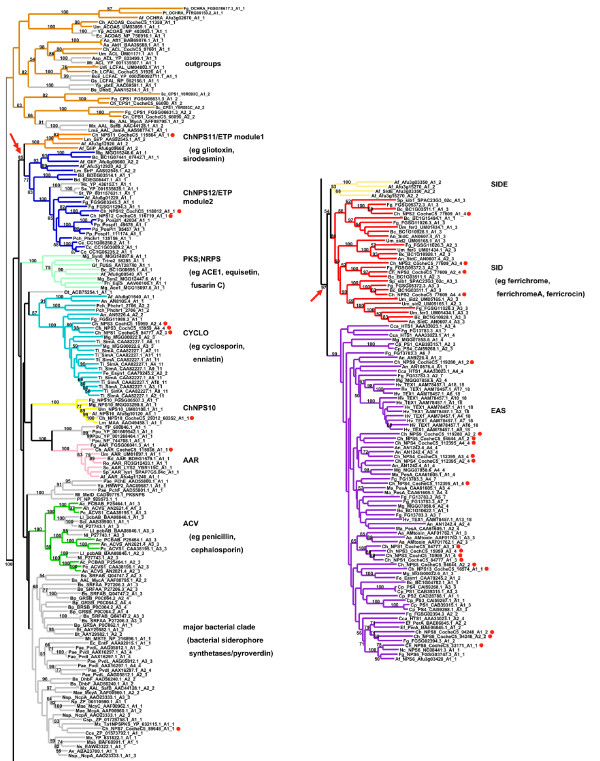
**ML phylogenetic tree (PhyML, WAG plus gamma) from the reduced A domain dataset**. Branches corresponding to subfamilies are color coded as in Fig. 1 and known products of NRPSs within each subfamily are shown to the right in parentheses. All *C. heterostrophus *NRPS A domains are indicated as red dots. Bootstrap values greater than 50% are shown above branches, where legibility makes this possible. This analysis shows stronger bootstrap support (97%) for grouping the exclusively fungal, multimodular subfamilies, SID and EAS subfamilies together (arrow). Double arrow indicates high bootstrap support (>85%) for grouping ChNPS11/ETP/ChNPS12 together.

All known NRPS/NRPS-like proteins formed a monophyletic group supported by greater than 90% bootstrap support in ML analyses and greater than 50% bootstrap support in the NJ analysis (Fig. 1), separating them from most other known adenylating enzymes selected as potential outgroups, e.g., Acyl AMP ligases (AAL), CPS1 [54], Long Chain Fatty Acid ligases (LCFAL), Acetyl-CoA synthetases (ACoAS), and Ochratoxin synthetases (OCHRA)(Fig. 1, Additional file 5). The α-aminoadipate reductases (AAR), homologs of *S. cerevisiae *Lys2 [23,55,57], grouped within this well-supported clade of NRPS/NRPS-like proteins rather than with the other adenylating enzymes (Figs. 1, 2, Additional file 6), suggesting that AARs are more closely related to NRPSs than to other adenylating enzymes.

The tree topologies resulting from phylogenetic analyses of individual A domains revealed two major groups of fungal NRPSs (Fig. 1, Additional file 6). The first group (Fig. 1, light blue rectangle) consists of primarily mono- or bi-modular fungal NRPSs which group with bacterial NRPS A domains. Exceptions to the predominately mono/bi-modular fungal NRPS structures include the ACV synthetases and the clade containing A domains from the eleven modules of SimA (cyclosporin biosynthesis) [58] and from several related fungal NRPSs. The other large group contains exclusively fungal and primarily multimodular NRPSs and includes siderophore synthetases and a group we term the Euascomycete-only synthetases, as its members are restricted to euascomycetes. Both grouped together with greater than 97% bootstrap support in analyses of a reduced dataset which included selected representatives from each subfamily (Fig. 2, red arrow, Additional file 7).

Phylogenetic analyses identified nine major subfamilies of fungal NRPSs. Subfamilies were defined as the most internal branch from the root node that formed a monophyletic group which was supported by greater than >70% bootstrap support, shared identical taxon composition across all three phylogenetic methods, and contained a representative fungal NRPS. These groups were named after a representative *C. heterostrophus *or other fungal NRPSs of well-known function in the group (Figs. 1, 2, Additional file 6). Subfamilies include: 1) fungal PKS;NRPS hybrids, 2) ChNPS11/ETP toxin module 1 synthetases, ChNPS12-like/ETP module 2 toxin-like synthetases, 4) ChNPS10-like synthetases, 5) Cyclosporin synthetases (CYCLO), 6) α-aminoadipate reductases (AAR), 7) ACV synthetases (ACV), 8) siderophore synthetases (SID), and 9) the Euascomycete-only synthetases (EAS). Deep phylogenetic relationships among mono/bi-modular subfamilies were unresolved and lacked bootstrap support (Figs. 1, 2, Additional file 6A-C). A domains from a few ascomycete (BC1G11613.1, MGG 14967.5, MGG07803.5) and several urediniomycete (UM05245.1, Sr31423, and PGTG06519.1) proteins did not group with any of the major subfamilies and were not placed consistently in the trees when assessed by different phylogenetic methods. Homologs of bimodular *A. fumigatus *SidE, a putative siderophore synthetase [37], formed two clades corresponding to each module and consistently grouped with the SID subfamily but without bootstrap support in the larger phylogeny and with low bootstrap support (>50%) in the reduced phylogeny. We term this group SIDE but do not consider them as a major subfamily (Fig. 2, Additional files 6A-C, 7)

### Relationships between fungal and bacterial NRPSs: horizontal transfer or vertical transmission and massive loss?

The majority of bacterial sequences (Additional file 8), identified as top hits in blast searches using a representative from each of the major fungal NRPS subfamilies to query the public databases, were eubacterial in origin and formed a monophyletic group (although lacking bootstrap support), which we term the major bacterial clade (Figs. 1, 2, gray, Additional file 6). This clade contains two fungal representatives suspected of being horizontally transmitted from bacteria to fungi. One is the fungal ChNPS7;PKS24 hybrid NRPS;PKS synthetase which is nested within this clade; previous independent analyses of both the NRPS [10] and the PKS portion of this protein [29] found the same placement (Fig. 2, Additional file 6). The other is the ACV synthetases, a group postulated to have been horizontally transferred from bacteria to fungi [59-64], which groups as sister to, or within, the major bacterial clade (Figs. 1, 2, Additional file 6). Our analysis also shows that each of the three fungal ACV synthetase A domains groups with the corresponding bacterial A domain rather than forming separate clades of fungal and bacterial A domains. These results support previous claims of horizontal transfer based on observations of closer sequence similarity than expected between these fungal and bacterial genes [61-64] (Fig. 2, Additional file 6).

In contrast, bacterial siderophore synthetases (eg. Pyoverdine (PvdD, PvdI, PvdJ, PvdL), yersiniabactin (ybtE), and Pyochelin (PchE, PchF)) group separately from fungal NRPSs (SID) that biosynthesize intracellular siderophores and fungal NRPSs (NPS6) in the EAS subfamily that biosynthesize extracellular siderophores (Fig. 2, Additional file 6). This suggests that fungal and bacterial capacities to chelate iron via small molecule siderophores have evolved independently (Fig. 2).

The remaining bacterial A domains included in this study that grouped with high bootstrap support with fungal A domains were associated with the ChNPS12-like/ETP module 2 and AAR subfamilies (Fig. 2, Additional file 6). Bacterial representatives of the former included representatives from the gammaproteobacterium (*Hahella chejuensis*) and two closely related species of actinobacteria (*Salinospora *sp.); of the latter, all are closely related species of *Pseudomonads*. In the two cases of proposed horizontal transfer discussed above (e.g., ChNPS7 [10,29], and ACV [59-64] synthetases), the fungal genes are nested within a large clade of bacterial sequences. The reverse phylogenetic situation is observed for bacterial genes grouping with the AAR and ChNPS12 subfamilies as, in these cases, bacterial NRPSs, are nested within a large clade of fungal NRPSs (ChNPS12) or group as sister to fungal NRPSs (AARs). These placements suggest that either the fungal genes were transferred to bacteria or that the origin of these groups predates the divergence of eukaryotes and prokaryotes and the observed pattern reflects extensive loss or incomplete sampling from bacteria. Clearly, further sampling of bacterial sequences is needed to adequately address these hypotheses, but we favor the theory that these NRPS subfamilies may have originated prior to the divergence of prokaryotes and eukaryotes. We hypothesize that the lack of phylogenetic signal for resolving relationships among the fungal mono/bi-modular subfamilies may in part reflect an ancient and rapid radiation of these groups.

### Distribution of NRPS subfamilies across fungal taxonomic groups

The distribution of fungal NRPS subfamilies across the major fungal taxonomic groups supports previous findings that NRPSs are much more abundant in Euascomycetes than in Basidiomycetes and are scarce in Chytridiomycota, Zygomycota, Schizosaccharomycota, and Hemiascomycota [10,65,66]. The number and distribution of NRPSs in each subfamily are shown in Table 1. EAS and PKS;NRPS subfamilies were significantly overrepresented in Euascomycete taxa when evaluated by Fisher's exact tests, while ChNPS12-like synthetases were statistically overrepresented in Basidiomycete taxa (Table 1, asterisks). The Chytridiomycota, Zygomycota, Schizosaccharomycota, and Hemiascomycota contained only a few NRPSs. All Zygomycota and Hemiascomycota lacked genes encoding NRPS-type proteins other than a single AAR. The chytrid genome contained two additional NRPS-like proteins grouping with the ChNPS12/ETP module 2 subfamily, and the two Schizosaccharomycota taxa examined contained one additional NRPS for siderophore biosynthesis (Table 1). No subfamilies were statistically overrepresented in these groups.

**Table 1:** Numbers of NRPS genes in each subfamily across fungal taxonomic groups^a^

Species	PKS; NRPS	NPS11/ETP mod 1	NPS12/ETP mod 2	NPS10	CYCLO	SID	ACV	AAR	EAS	Other^b^	Total
**Ascomycota**	*								*		

*A. fumigatus*	1	2^c^	1	1	1	1	0	1	10	2	20
*A. nidulans*	1	0	1	1	2^d^	1	1	1	11	0	19
*B. cinerea*	3^e^	1	0	0	0	3	0	1	5	1	14
*C. immitis*	1	0	0	0	0	1	0	1	5	0	8
*C. heterostrophus*	0	1	2	1	2^d^	1	0	1	6	1	15
*F. graminearum*	1	0	3	1	1	2	0	1	12	0	21
*M. oryzae*	6	1	1	1	1^d^	1	0	1	4	2	18
*N. crassa*	0	0	0	0	0	1	0	1	2	0	4
*P. anserina*	4	0	1	0	0	1	0	1	4	1	12
*T. reesii*	2	2^c^	1	1	0	1	0	1	5	0	13
											
**Basidiomycota**			*								
*C. cinerea*	0	0	3	0	0	1	0	1	0	0	5
*C. neoformans*	0	0	0	0	0	0	0	1	0	0	1
*L. bicolor*	0	0	0	0	0	0	0	1	0	0	1
*P. chrysosporium*	0	0	1	0	1	0	0	1	0	0	3
*P. stipitis*	0	0	0	0	0	0	0	1	0	0	1
*P. placenta*	0	0	8	0	0	0	0	2	0	0	10
*P. graminis*	0	0	0	0	0	0	0	1	0	1	2
*S. roseus*	0	0	0	0	0	0	0	1	0	1	2
*U. maydis*	0	0	0	1	0	2	0	1	0	1	5
											
**Schizosaccharomycota**											
*S. japonicus*	0	0	0	0	0	1	0	1	0	0	2
*S. pombe*	0	0	0	0	0	1	0	1	0	0	2
											
**Hemiascomycota**											
all species examined (Additional file 3)	0	0	0	0	0	0	0	1	0	0	1
											
**Zygomycota**											
*P. blakesleeanus*	0	0	0	0	0	0	0	1	0	0	1
*R. oryzae*	0	0	0	0	0	0	0	1	0	0	1
											
**Chytridiomycota**											
*B. dendrobatidis*	0	0	2	0	0	0	0	1	0	0	3
											
**Microsporidia**											
*E. cuniculi*	0	0	0	0	0	0	0	0	0	0	0

^a^Based on inclusion in clades of phylogenetically defined subfamilies (Fig. 1, Additional file 6).

^b^Several proteins which grouped with NRPSs did not group with any of the 9 major subfamilies. These include homologs of SidE (Afu3g03350) in *A. fumigatus *and *P. anserina *species (Afu3g15270, Pa2_7870), several proteins in the urediniomycetes *U. maydis, S. roseus, and P. graminis *(UM05245.1, Sr31423, and PGTG06519), two proteins in *M. oryzae *(MGG 14967.5, MGG07803.5), and one in *B. cinerea *(BC1G11613.1).

^c^*A. fumigatus *has two bi-modular NRPSs (Afu_6g09660, Afu_6g09660) as does *T. reesii *(Trire2_24586, Trire2_60458). The first modules of all four NRPSs group with the ChNPS11/ETP module 1 subfamily; the second modules group with the NPS12 subfamily. For tallying purposes, Afu_6g09660, Afu_6g0966, Trire2_24586, and Trire2_60458 were attributed to the ChNPS11/ETP module 1 subfamily.

^d^ChNPS1 and ChNPS3 modules 1 and 3 group with the EAS subfamily, while ChNPS1 module 2 and ChNPS3 modules 2 and 4 group with the CYCLO subfamily. For tallying purposes, ChNPS1 and ChNPS3 were attributed to the CYCLO subfamily. Similarly, MGG00022.1 and AN9226 also have some A domains in the CYCLO subfamily and others in the EAS subfamily. For tallying purposes, these genes were included in the CYCLO subfamily.

^e^*B. cinerea *contains 3 PKS;NRPS hybrids. For one of these (BC1G15479.1), the A domain did not align well and was missing several core motifs. This protein was removed from the final phylogenetic analysis.

* PKS:NRPS and EAS subfamilies in Euascomycetes and the NPS12/ETP module 2 subfamily in Basidiomycetes are statistically over-represented.

#### Lineage specific expansions and contractions

**Figure 3:**
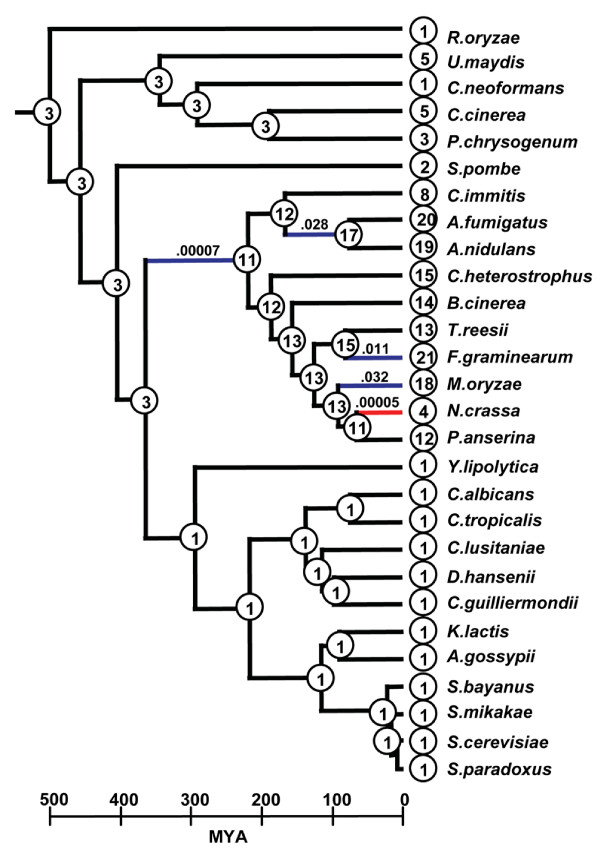
**Lineage specific expansions and contractions in number of NRPS genes per genome**. Inferred number of NRPS encoding genes at ancestral nodes mapped onto the ultrametric tree of fungi. Timescale in millions of years is shown along bottom. Branches with significant expansions (blue) or contractions (red) are shown with associated p-values above branches. The largest contraction in number of NRPSs occurs in *N. crassa *while the largest expansion occurs in the ancestor of euascomycetes. A highly significant expansion also occurs in *F. graminearum *and significant expansions occur in several other euascomycete taxa (e.g., *M. oryzae *and on the branch leading to the *Aspergillus *species).

When patterns of gene duplication and loss were analyzed for the total number of NRPSs/genome (combining all subfamilies) over the tree of fungi (Fig. 3; Additional file 9), a highly significant expansion was found on the branch leading to Euascomycetes (p = 7 × 10^-5^). Significant expansions were also found within euascomycetes on the branches leading to the *Aspergillus *species (p = .028), to *F. graminearum *(p = .011) and to *M. oryzae *(p = .032). *N. crassa *showed a highly significant (p = 5 × 10^-5^) contraction in total number of NRPS genes (Fig. 3), likely due to the efficiency of RIP and/or other genome defense mechanisms, that reduce the rate of fixation of duplicates [67,68].

Our data support previous findings [66], including our own [10], that unicellular fungi have few, if any, genes for secondary metabolism (Table 1, Fig. 3). Ancestral reconstructions show that in hemiascomycete yeasts, this is due to loss of all NRPSs, except for a single AAR encoding gene, that were present in basidiomycetes and inferred to be present in the ancestor of ascomycetes (Fig. 3). However, both the fission yeast *S. pombe *and the unicellular basidiomycete yeast *Sporobolomyces roseus *contain one additional gene encoding a NRPS (a siderophore synthetase and an unknown, respectively) in additional to the single AAR encoding gene, suggesting that a unicellular habit may not preclude the existence of secondary metabolite genes such as NRPSs. Patterns of expansion and contraction also do not seem to occur preferentially in fungal pathogens *versus *nonpathogens. While a number of pathogenic fungi (e.g., *F. graminearum, A. fumigatus*, and *M. oryzae*) do show evidence for expansions in numbers of NRPS, we also see expansion in the nonpathogen, *A. nidulans*.

#### Subfamily distribution

##### AAR

A single ortholog of *S. cerevisiae *Lys2, the AAR involved in reduction of α-aminoadipic acid in the fungal lysine biosynthetic pathway [23,69], was found in all fungi surveyed except the Microsporidian, *Enchephalitozoon cuniculi*, an intracellular parasite which has lost the majority of genes involved in amino acid biosynthesis [70] and the heterokaryotic basidiomycete *Postia placenta*, which contains two (Table 1)(Additional file 2).

##### ChNPS11/ETP/ChNPS12

In a phylogeny of a reduced set of representative A domains from each subfamily (Fig. 2), homologs of ChNPS11, ChNPS12, and the ETP toxin synthetases, GliP for Gliotoxin and SirP for Sirodesmin production, group together with strong bootstrap support (>80%), suggesting all share a common evolutionary origin. In the larger phylogeny of the complete dataset (Fig. 1, Additional file 6), they formed two separate clades each supported by >70% bootstrap support, but lacked this level of support for the entire group. The first clade (ChNPS11/ETP module 1) includes the first module of the ETP toxin synthetases and monomodular ChNPS11. The second module of the ETP toxin synthetases, however, groups within a larger clade containing the two NRPSs from the chytrid genome, several eubacterial NRPSs, and a clade containing both euascomycete and basidiomycete homologs of ChNPS12 (ChNPS12/ETP module 2). While fungal NRPSs associated with ChNPS11 and ETP toxin synthetases are found only in Euascomycota, NRPSs from both eubacteria and from the most basal fungal group, Chytridiomycota, were nested within this larger clade with high bootstrap support (>80%) (Figs. 1, 2).

##### ChNPS10, CYCLO, SID

Three subfamilies, monomodular ChNPS10, NRPSs grouping with SIMA (CYCLO), and NRPSs (SID) involved in intracellular (primarily) siderophore biosynthesis, contain representatives from both Basidiomycota and Euascomycota. While all euascomycetes and many basidiomycetes examined contain at least one representative from the SID subfamily (Table 1) [65], ChNPS10 and CYCLO are more discontinuously distributed and a representative is not found in all taxa (Table 1, Additional file. 6).

##### ACV and PKS;NRPS

PKS;NRPSs were restricted to and statistically overrepresented in euascomycetes. As has been noted previously [29,71], all fungal PKS;NRPS hybrids fall into a single, well supported, monophyletic group, which suggests a single origin (Table 1). However, not all ascomycetes have a representative of this group and the number of corresponding genes varies widely among taxa (Table 1). *C. heterostrophus*, for example, lacks a representative but *M. oryzae *has six. While ACV synthetases are found in both bacteria and fungi, within fungi, they appear restricted to Eurotiomycete and Hypocrealean taxa. This study did not identify any additional ACV synthetases in fungi apart from the known ones in *Penicillium chrysogenum*, *Aspergillus nidulans*, and *Cephalosporium acremonium *(Additional files 2, 6), supporting previous conclusions that their distribution is likely the product of one or more isolated horizontal transfer events [59,61-64].

##### Euascomycete only (EAS)

The EAS subfamily contains by far the greatest number of NRPSs and is both restricted to and statistically overrepresented in Euascomycetes (Table 1).

### Hypothesized origins based on taxonomic distribution

**Figure 4:**
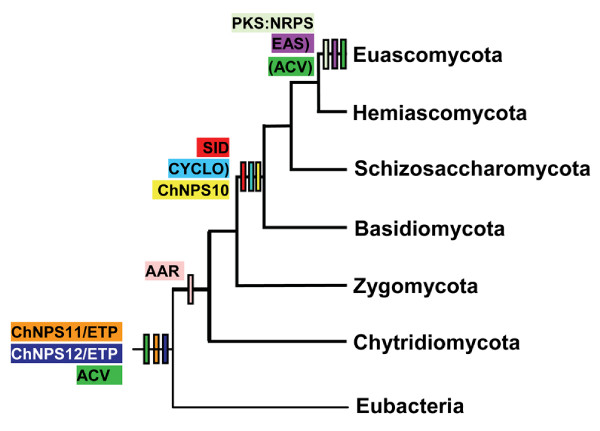
**Hypothesized origins of major fungal NRPS subfamilies based on the oldest member of each subfamily**. Subfamilies color coded as in Fig. 1. AAR, and ChNPS11/ETPmodule 1 and ChNPS12/ETP module 2 likely originated prior to or early in the divergence of fungi. AAR genes are present in all fungi, while the ChNPS11/ETP/ChNPS12 clade contains representatives of the most ancestral fungal group, the Chytridiomycota. as well as bacterial sequences that nest with high bootstrap support within this clade. Although ACV genes are clearly present in eubacteria, they appear to have been horizontally transferred to euascomycete fungi, hence their dual placement. The CYCLO, ChNPS10, and SID subfamilies were found in Basidiomycota, Schizosaccharomycota, and Euascomycota and thus likely originated in an ancestor of the Dikarya. Fungal PKS;NRPS hybrids and EAS were found only in Euascomycetes.

Fig. 4 shows the hypothesized origins of each subfamily based on taxonomic distribution of the oldest member of each group. By this criterion, the presence of bacterial sequences grouping within the ChNPS11/ETP module 1 and ChNPS12/ETP module 2 clades suggests that the origins of these groups may predate the divergence of eubacteria and eukaryotes (Figs. 2, 4). The AAR subfamily must have arisen also either prior to or very early in the origin of the fungi as a representative is present in all fungi, including the most basal group, the Chytridiomycota (Table 1, Figs. 2, 4). Since the SID, CYCLO, and ChNPS10 subfamilies all contain representatives from both Euascomycota and Basidiomycota, these groups must have evolved prior to the divergence of the Dikarya (Fig. 4). The EAS, PKS;NRPS, and ACV synthetases contained only euascomycete representatives. Both PKS;NRPS and EAS may thus have originated in the ancestor of euascomycetes (Fig. 4). As discussed above, the grouping of fungal ACV synthetase A domains with the corresponding A domains of bacterial ACV synthetases within a large clade of bacterial sequences provides evidence for horizontal transfer and suggests that this group originated within prokaryotes (Fig. 4).

Thus, taxonomic distributions suggest a more ancient origin of one or more of the mono/bi-modular NRPS subfamilies (ChNPS11/ETP/ChNPS12, ACV), possibly predating the divergence of eubacteria and fungi (Table 1, Fig. 4). The strongly supported co-grouping of fungal and bacterial sequences in the ChNPS11/ETP/ChNPS12 group, as in the outgroup adenylating enzymes (Fig. 2, Additional file 6A-C) suggests this is a tenable hypothesis. ACV is a special case and likely the result of horizontal transfer from bacteria to fungi. In contrast, the fungal-specific multimodular groups (SID and EAS), which group together with high bootstrap support in the reduced phylogeny (Table 1, Fig. 2, Additional file 6A-C), appear to be of more recent origin and are restricted to and highly expanded in fungi.

### Mono- and bi- modular NRPS subfamilies

**Figure 5:**
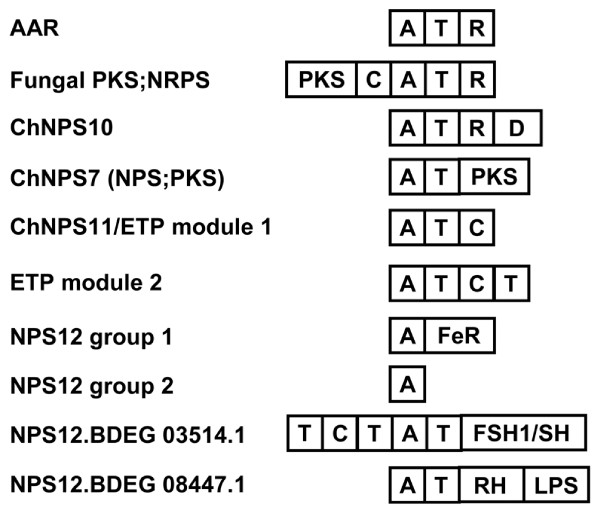
**Conserved domain architectures for mono-bimodular NRPS subfamilies**. The majority of mono-bimodular subfamilies have an A-T domain structure followed by various C-terminal domains. Only ChNPS11/ETP module 1 and ETP module 2 show complete A-T-C modules. The ChNPS12/ETP module 2 subfamily also contains representatives consisting of a single A domain. Domains: A = adenylation, T = thiolation, C = condensation, R = thioester reductase, D = ADH short chain dehydrogenase, PKS = polyketide synthase module, FeR = ferric reductase, FSH/SH = serine hydrolase, RH = polynucleotidyl transferase, Ribonuclease H, LPS = LPS-induced tumor necrosis alpha factor.

Unlike many of the multimodular NRPSs, most monomodular subfamilies lack a complete NRPS module (A-T-C) and consist of a single A domain or an A-T domain combination followed by a variety of C-terminal domains (Fig. 5). Many of the mono/bi-modular groups show a conserved domain architecture across all members in a subfamily, suggesting their domain architectures may be functionally constrained. Available functional data suggest that the NRP products of several of these groups may play more central roles in cellular metabolism related to responses to oxidative stress and growth and development.

Whether monomodular NRPSs may act alone or in concert with non-NRPS proteins is currently unknown. However, in bacterial systems, both single A domains as well as A-T domain units, known as initiation modules, can interact with other NRPS proteins and accomplish biosynthesis by first activating and then transfering the activated substrate either to a C domain in the same NRPS or to a C domain in a different NRPS (nonlinear biosynthesis) [5].

#### AARs and Lysine biosynthesis

AARs are conserved not only taxonomically but also in terms of domain structure. All have an identical structure consisting of an A-T unit followed by a thioester reductase (R) domain (IPR010080), a member of the NAD(P)-binding Rossman fold domain superfamily (SSF51735). There are two primary pathways for lysine biosynthesis, the diaminopimelic acid pathway (DAP), found predominantly in bacteria and plants, and the α-aminoadipate pathway (AAA), found primarily in fungi and a few bacteria [69]. As noted above, AARs catalyze reduction of α-aminoadipic acid in the AAA pathway [69]. The fact that AARs have a C-terminal R domain in common with several other NRPS subfamilies (PKS;NPRS, ChNPS10, EAS, discussed below) supports our conclusions based on phylogenetic relationships that AARs are more closely related to NRPSs than other adenylating enzymes (Fig. 5).

Bacterial sequences grouping with fungal AAR are comprised of a single A domain followed by an acyl-transferase domain (PFAM01757) but lack the C-terminal R domain found in fungal AARs. We conclude that they are likely not involved in lysine biosynthesis in bacteria. Although there is evidence for the existence of lysine biosynthesis through the AAA pathway in some prokaryotes [72], current data suggests that these pathways do not include a step involving reduction of α-aminoadipic acid [72]. Thus, our data support previous conclusions that AARs are fungal-specific enzymes [73-75].

#### PKS;NRPS

Nearly all fungal PKS;NRPS hybrids have the same domain structure (KS-AT-M-KR-ACP-C-A-T-R) (Fig. 5, Additional file 2). The terminal R domain has been reported previously in several PKS;NRPS hybrids [76-78].

#### ChNPS10

The ChNPS10 subfamily also has a conserved domain architecture across all genes in the subfamily, consisting of an A-T unit followed by two additional C-terminal domains. The first is a NAD(P) binding domain (IPR016040) also showing closest similarity to thioesterase reductase (R) domains and the second is a dehydrogenase domain with closest hits to ADH short chain dehydrogenases (IPR002198) (Fig. 5).

#### ChNPS11/ETP/ChNPS12

**Figure 6:**
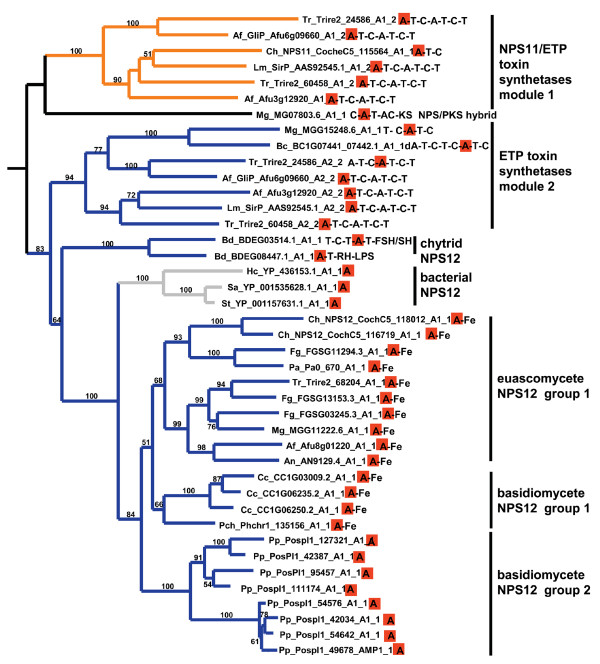
**Phylogeny of the ChNPS11/ETP/ChNPS12 subclade**. Extracted from maximum likelihood (PhyML with WAG plus gamma substitution matrix) phylogeny of complete A domain dataset (Additional file 6B). Domain structure of each NRPS is shown to the right of species abbreviation and accession number. Orange highlighted A domains reflect corresponding A domain in the phylogeny. Orange branches = ChNPS11/ETP mod1 and blue = ChNPS12/ETP mod2 subfamilies. ChNPS11 is monomodular, while all other NRPSs in the ETP module 1 group are bimodular; all have complete A-T-C modules. The A domain from a *M. oryzae *NRPS;PKS (MG07803.6) also groups here. Members of the ChNPS12 subfamily show a diversity of C-terminal domains as described in text and Fig. 5. The group includes two putative NRPSs from the chytrid, *B. dendrobatidis*, two proteins with either an incomplete (MGG15248.6) or a degenerate (BC1G07441_07442.1) first module and monomodular bacterial proteins consisting of single A domains. ChNPS12 homologs in Basidiomycete NPS12 group 2 consist of proteins with single A domains which appear to lack additional C-terminal domains and are highly expanded in the basidiomycete *Postia placenta*.

The large and highly diverse clade of ChNPS11/ETP/ChNPS12 homologs reveals the diversity of C-terminal domains that can follow A-T units and shows that, as for some bacterial NRPSs, fungal NRPS or NRPS-like proteins can consist of single A domains (Figs. 5, 6).

At the base of this group are monomodular ChNPS11 and module 1 of the bimodular ETP toxin synthetases, SirP and GliP, which contain complete A-T-C modules (Fig. 6). Module 2 of SirP and GliP groups at the base of the ChNPS12/ETP module 2 clade. The second module of the ETP toxin synthetases contains a complete module followed by an additional T domain (A-T-C-T) (Fig. 6). This group also contains several fungal proteins with an incomplete (MGG15248.6) or a degenerate (BC1G07441_07442.1) first module (Figs. 2, 5).

Nested within this clade is a group of bacterial NRPSs with a single A domain and two NRPS-like proteins from the chytrid *B. dentrobatidis *(Fig. 6). One of the chytrid NRPSs (BDEG_03514.1) has a T-C-T-A-T domain architecture followed by a domain with similarity to FSH1 (IPR005645), a serine hydrolase domain. The other chytrid protein (BDEG_08447.1) has an A-T unit followed by two additional domains. The first shows closest similarity to polynucleotidyl transferase, Ribonuclease H fold (IPR012337), a domain associated with nucleic acid binding functions and found in a variety of proteins including HIV RNase H, transposases, and exonucleases [79,80] (Fig. 6). The second domain shows closest similarity to the membrane-associated domain LPS-induced tumor necrosis factor alpha factor (LITAF, IPR006629, PF10601), which contains a characteristic cysteine rich zinc-binding motif found also in intracellular Zn^2+ ^binding proteins and animal transcription factors. The zinc and DNA-binding domains found in the chytrid NRPSs are intriguing (Fig. 5). Gliotoxin and Sirodesmin PL have been shown to inhibit viral reverse transcriptase [81] and general transcription [82], respectively. In the case of Sirodesmin PL, the addition of zinc and other IIB series metals (Hg and Cd) both decreases toxin production in *Leptosphaeria maculans *and also reverses the inhibition of transcription, suggesting interactions of Sirodesmin PL with either cellular zinc or zinc-containing metalloenzymes such as RNA polymerases [82,83]. Whether these phenotypes relate to our identification of Zn-binding domains in the corresponding chytrid NRPS is unknown.

ChNPS12 (CocheC5_118012), and its paralog (CocheC5_116719) contain a single A domain followed by a domain showing closest similarity to a ferric reductase transmembrane domain (IPR013130). The closest homologs of ChNPS12 (Fig. 6, euascomycete ChNPS12 group 1) are present in both euascomycete and basidiomycete group 1 and have the same domain structure as the *C. heterostrophus *NPS12 proteins (Fig. 6). Sister to all group 1 NPS12-like proteins is a group of proteins consisting of standalone A domains (Fig. 6, basidiomycete NPS12 group 2). These were found only in the brown-rot heterokaryotic fungus, *P. placenta*, which carries eight closely related copies.

The monomodular bacterial NRPSs nested within the ChNPS12/ETP module 2 subfamily also consist of a standalone A domains. As noted earlier, for many bacterial NRPS systems (e.g., VibE, MxcE, and YbtE), single A domains may be involved in NRPS biosynthesis by activating and transferring the activated substrate to a different NRPS [5]. Only one example of this type of synthesis has been reported for fungi (e.g., *C. purpurea *ergot alkaloid biosynthesis) [5,84], but our identification of these single fungal A domains grouping with other known NPRSs (e.g., ETP toxins) (Figs. 2, 6, Additional file 6) suggests that this mechanism could be more common in fungi than previously appreciated.

The diversity of domain structures found within the ChNPS11/ETP/ChNPS12 group leads us to hypothesize that there may be several distinct functional groups within this clade.

### Multimodular NRPS subfamilies

The majority of multimodular NRPSs are found in the SID and EAS subfamilies. These subfamilies group together with high bootstrap support (>97%) in analyses of the reduced dataset (Fig. 2). Analyses that included a larger number of bacterial sequences (KE Bushley and BG Turgeon, unpublished) support our phylogenetic and distribution data that the SID and EAS subfamilies are restricted to fungi. As noted above, two subfamilies containing genes encoding multimodular NRPSs, the CYCLO and ACV synthetases, group with the primarily mono/bi-modular suite of NRPSs. (Table 1, Fig. 2). SID synthetases show a relatively conserved domain architecture, are present in the majority of euascomycetes sampled, and are thought to have evolved by module duplication and selective loss of A domains or complete modules, as described in detail in Bushley et al. [65].

#### Diversity within the EAS subfamily

**Figure 7:**
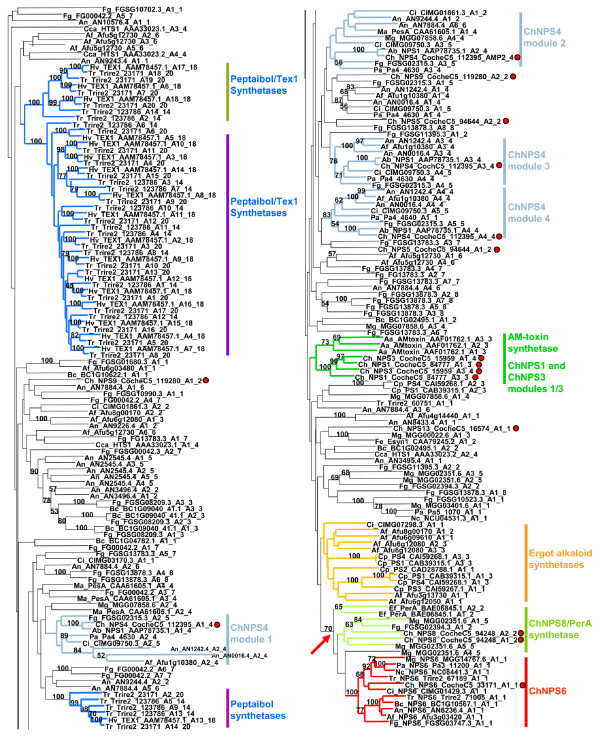
**Phylogenetic analysis of the Euascomycete subclade**. Tree extracted from the maximum likelihood (PhyML with WAG plus gamma substitution matrix) phylogeny of the complete A domain dataset (Additional file 6B). Branches defining subgroups of the EAS clade grouping with a *C. heterostrophus *NRPS A domain or with A domains from fungal NRPSs with known function are color coded: dark blue = peptaibol synthetases, light blue = ChNPS4 (clades grouping with each A domain of *C. heterostrophus *NPS4), green = AMT synthetases and ChNPS1 and ChNPS3 modules 1 and 3, orange = ergot alkaloid synthetases, light green = ChNPS8/PerA synthetases, and red = homologs of ChNPS6 (extracellular siderophore synthetases). Of these groups, only the peptaibol synthetases, the clade containing NPS8/PerA/NPS6 synthetases (arrow), and ChNPS4 modules 3 and 4 have bootstrap support >70%. *C. heterostrophus *NRPS A domains are indicated (circles).

The EAS subfamily, in additional to containing the vast majority of fungal NRPSs, also shows the greatest diversity of both domain architecture and function (Figs. 2, 7, Additional file 2). It includes proteins that are both structurally and functionally conserved (e.g. homologs of ChNPS6 which biosynthesize extracellular siderophores), as well as those that are highly lineage specific (e.g. HTS1 [51] and AMT [52] synthetases for host selective toxins, Tex1 [85] and other peptaibol synthetases, and ergot alkaloid synthetases). The highly diverse domain architectures and discontinuous distribution of corresponding A domains make the identification of orthologs across species extremely challenging.

##### ChNPS6/PerA

Perhaps the only group for which orthologs can be clearly identified are homologs of the most conserved NRPS in the EAS clade, ChNPS6, which biosynthesizes an extracellular iron scavenging siderophore that serves as a virulence factor for several fungi and is also involved in combating oxidative stress [10,6] (Figs. 2, 6, 7). Although ChNPS6 appears to have undergone a gene duplication event, it is single copy in all species examined except *Trichoderma reesii *(Fig. 7), which contains two paralagous copies. All ChNPS6 homologs have a highly conserved domain structure consisting of a single A-T-C module followed by a module with a degenerate A domain (dA-T-C) [10]. Sister to the ChNPS6 group is a clade containing both ChNPS8 and an *Epichloe festucae*NRPS, PerA; the latter NRPS mediates symbiotic interactions of *E. festuca *with its grass host by producing an NRP insect deterrent, peramine [86] (Fig. 7, arrow).

##### Ergot alkaloid synthetases

NRPSs synthesizing ergot alkaloids consistently grouped sister to the ChNPS6 and ChNPS8/PerA clade but without bootstrap support (Figs. 2, 7). These synthetases were found only in animal pathogens in the Eurotiales and grass endophytes such as *C. purpurea *(Figs. 2, 7). Given that grass endophytes such as *C. purpurea *are thought to have an animal pathogenic ancestor [87] and that their ergot alkaloid NRP products have toxic effects on livestock and other animals [88-91], we hypothesize that NRPSs synthesizing ergot alkaloids originally evolved to function in animal pathogenesis.

##### Peptaibol synthetases

Peptaibol synthetases, which were restricted to the Hypocrealean taxa examined in this study (*Trichoderma/Hypocrea*), also formed a well supported group. However, as discussed below, several modules of each peptaibol synthetase group outside of the main clade (Table 1, Figs. 2, 7)

##### Dothideomycete host-selective toxin synthetases

A domains of the *A. alternata *apple pathotype-specific AMT synthetase which produces the host-selective toxin, AM toxin, grouped consistently with modules 1 and 3 of ChNPS1 and ChNPS3 (discussed below). Modules of tetramodular *C. carbonum *HTS1, responsible for biosynthesis of another host selective toxin, the cyclic tetrapeptide, HC-toxin, grouped in disparate locations in the EAS clade such that clear homologs of HTS1 A domains were not recognizable in any of the species in our dataset (Figs. 2, 7).

##### ChNPS4

Both HTS1 and ChNPS4 A domain relationships exemplify the challenges of identifying orthologs within the rapidly evolving EAS clade. ChNPS4 has been shown to play a role in *C. heterostrophus *conidial cell surface hydrophobicity and a homolog in the related Dothideomycete *Alternaria brassicicola *plays a role in conidial wall development and integrity [9]. Each of the four A domains from each module of tetramodular ChNPS4 groups with strong support with the corresponding A domains of tetramodular AbNPS1 in the closely related Dothideomycete, *A. brassicae*. These A domains group within a larger clade containing *Metarhizium anisopliae *NRPS PesA although without bootstrap support (Figs. 2, 7, Additional file 6). However, A domains from NRPSs found in other euascomycetes that group with each of the ChNPS4 modules contain from two to six modules. While some of these A domains are clearly related to those of ChNPS4, module duplication and loss obscure the history of this group.

### Evolutionary mechanisms giving rise to multimodular NRPSs

The greater diversity of domain architectures seen in multimodular NRPSs is likely due to the multiplicity of evolutionary mechanisms which may generate the corresponding multimodular genes. The EAS subfamily, in particular, contains NRPSs varying from monomodular proteins involved in ergot alkaloid biosynthesis (PS2 and PS4) and ChNPS6 (which has one complete and one degenerate A domain) to the eighteen module TEX1 synthetase responsible for peptaibol biosynthesis in *Trichoderma virens *(*Hypocrea virens*) [85] (Figs. 2, 7, Additional file 6). Several subgroups within the EAS illustrate some of the mechanisms by which the diverse domain architectures of multimodular NRPSs may arise.

#### Tandem Duplication

Cyclosporin synthetase (SimA) is a clear example of tandem duplication of modules of an NRPS in a single species (*Tolypocladium inflatum*). All eleven A domains from this protein group together as a single well supported monophyletic group (Fig. 2) which also includes certain A domains from other fungal NRPSs, such as ChNPS1 module 2 and ChNPS3 modules 2 and 4.

**Figure 8:**
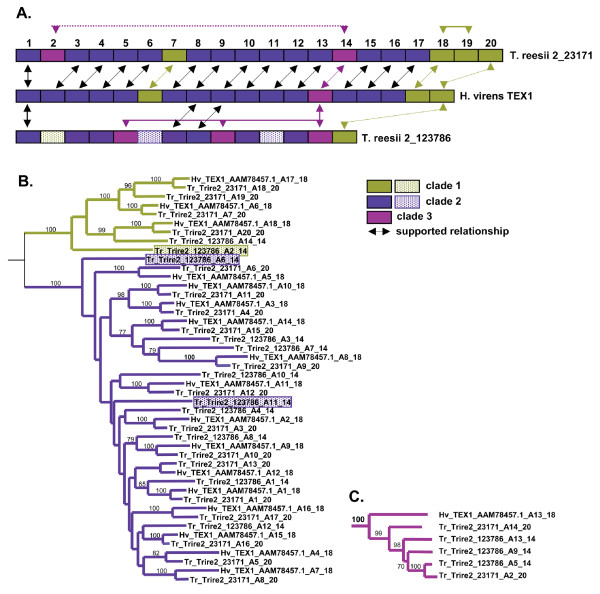
**Modular organization of Peptaibol synthetases and proposed evolution by tandem duplication**. A domains from peptaibol synthetases form three distinct, well-supported clades in the EAS subfamily (Fig. 7). **A**. Modular structure of the *H. virens *TEX 1 peptaibol synthetase and two peptaibol synthetases in the related species, *T. reesii *(T.reesii 2_23171 and T.reesii 2_123786. Color coding corresponds to clades identified in phylogenetic analyses (B and C, and Fig. 7). Arrows indicate bootstrap support for module relationships (B, C. and Fig. 7). While T. reesii 2_23171 is clearly a homolog of TEX1, domain duplication of modules 18 to 19 or vice versa and addition of module 2 have occurred since the common ancestor of these species. **B**. Two of the peptaibol synthetases clades (light green and dark blue, Fig. 7), group together as a monophyletic group but without bootstrap support. A domains shown in stippled boxes indicate modules from T.reesii 2_123786 which do not have a clear counterpart in the other peptaibol synthetases and may represent ancestral domains. **C**. The third clade (purple, Fig. 7) groups in a distinct position within the EAS subtree.

Peptaibol synthetases illustrate a more complex process of tandem duplication of modules of an NRPS. Peptaibol synthetases are highly lineage specific and found only within the Hypocreales to date. Using *H. virens *TEX1 as a point of reference, we found that all modules of TEX1 group together in three separate, well-supported clades with modules of two peptaibol synthetases (Trire2_23171 and Trire2_123786) in the related species, *Trichoderma reesii *(Figs. 2, 7). TEX1 module 13 falls outside of the other two TEX1 clades (Figs. 2, 7, 8), The nearly one-to-one relationship between modules of TEX1 and modules of *T. reesii *Trire2_23171 suggests that tandem duplication of modules giving rise to these orthologous genes must have occurred prior to divergence of these two species (Fig. 8). However, at least one additional internal duplication has occurred since divergence from an ancestral species (e.g., note the relationship between *T. reesii *Trire2_23171 modules 18 and 19) (Fig. 8). The relationship of these two peptaibol synthetases with the *T. reesii *14 module peptaibol synthetases, Trire2_123786 is less straightforward. However, we note that certain A domains from Trire2_123786 modules 2, 6, and 11 form widowed branches at the base of clades which contain A domains of at least two, and more often, all three peptaibol synthetases (Fig. 8, stippled boxes). We hypothesize that these may be ancestral domains. Previous studies suggest that like *T. reesii*, *T. virens *also harbors additional NRPSs involved in peptaibol biosynthesis [92].

#### Recombination

**Figure 9:**
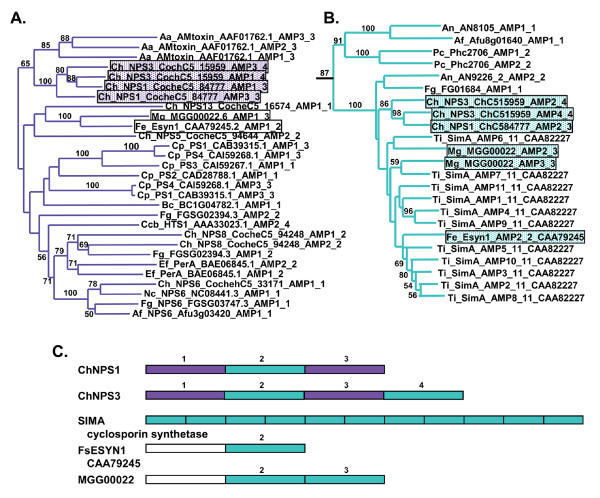
**Phylogenetic groupings and modular organization of ChNPS1 and ChNPS3 showing recombinant structure of these NRPSs**. **A**. Modules 1 and 3 of both ChNPS1 and ChNPS3 group with AM toxin synthetase, a trimodular NRPS that biosynthesizes AM-toxin, an *Alternaria alternata *host-selective toxin. **B**. Module 2 of ChNPS1 and modules 2 and 4 of ChNPS3 group with A domains (SimA) of cyclosporin synthetases (CYCLO) in a disparate position in the larger phylogeny compared to modules 1 and 3 (**A**, above) which group in the EAS subfamily (Fig. 2). **C**. Recombinant domain organization of ChNPS1 and ChNPS3. Blue boxes correspond to modules 2 and 4, purple boxes to modules 1 and 3. Note that single modules homologous to these domains are found in other euascomycete NRPSs. For example, Enniatin synthetases (Esyn1) and MGG00022.6 are also recombinant like ChNPS1 and ChNPS3 with one or more modules grouping with the cyclosporin subfamily (blue boxes) and others also within the EAS subfamily but in a distinct position from the ChNPS1 and ChNPS3 modules (clear boxes). Cyclosporin synthetases itself appears to have arisen by tandem duplication of SimA modules within *T. inflatum*.

Two NRPSs found in *C. heterostrophus*, ChNPS1 and ChNPS3, demonstrate the potential role of recombination and modular rearrangement in the generation of multimodular NRPSs. Modules 1 and 3 of both ChNPS1 and ChNPS3 group within the EAS subfamily with AMT synthetase, a lineage specific NRPS found only in a single strain of related *A. alternata *[52] (Figs. 2, 7, Additional file 6A-C). Module 2 of ChNPS1 and modules 2 and 4 of ChNPS3, however, group with the CYCLO synthetases among the mono/bi-modular NRPS subfamilies (Fig. 2, Additional file 6). The phylogenetically unlinked locations of ChNPS1 and ChNPS3 modules in the larger phylogeny suggests that a recombination event must have given rise to the extant genes in *C. heterostrophus *(Fig. 9). A domains of several other euascomycete NRPSs, for example, bimodular *Fusarium equiseti *Enniatin synthetase (FeESYN1) and trimodular *M. oryzae*, MGG00022, also show recombinant structures. Module 1 A domains of both proteins group in the EAS clade with the *C. heterostrophus *pseudogene ChNPS13, but without bootstrap support (Fig. 2), at positions distinct from modules 1 and 3 of ChNPS1 and ChNPS3. The C-terminal A domain of ESYN1 and the A domains of the final two modules of MGG00022 group in the CYCLO clade (Figs. 2, 9), like module 2 of ChNPS1 and modules 2 and 4 of ChNPS3. Thus, homologs of modules of ChNPS1 and ChNPS3 appear in different combinations in other fungi and demonstrate that recombination plays an important role in the evolution of multimodular NRPSs.

### Stability of NRPS gene copy number and domain architectures across subfamilies

Many multigene families experience gene duplication and loss and evolve by a birth-death process [93-96]. Variation in gene copy number resulting from gene duplication and loss is thought to be influenced by both functional and dosage requirements as well as random processes such as genomic drift [43,44,97,98]. Recent studies suggest that functionally conserved genes, such as those involved in growth and development or other basic cellular processes, tend to experience both less variation in copy number [53] and more stable domain organizations [49] than genes involved in environmental and stress responses [53,99].

For multimodular genes such as NRPSs, duplication and loss or birth-death evolution [93-95] can occur at two hierarchical levels: 1) at the level of the whole gene, and 2) at the level of domains within a gene (intragenic). In the latter case, genes encoding NRPSs whose products are involved in more conserved functions, such as the AARs, would be expected to have more stable domain architectures than those encoding proteins with niche-specific functions. The latter may experience less functional constraint allowing for flexible gain and loss of domains leading to diversity of domain structures. Because NRPS A domains are involved in substrate selection [100,101], their loss or gain could result in a rapid change in the chemical product of an NRPS.

**Figure 10:**
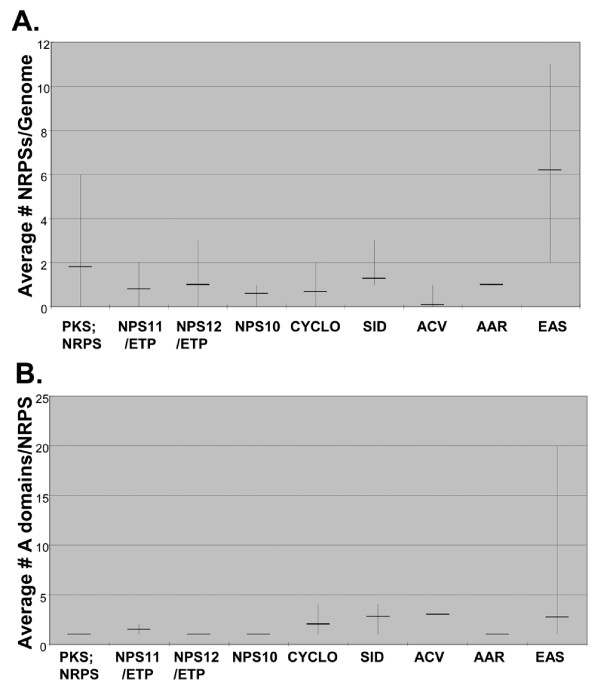
**Number and range of NRPSs and A domains for each subfamily**. **A**. Average and range (lowest to highest) number of NRPS-encoding genes in each subfamily per euascomycete genome shows that the EAS subfamily has both the highest average number of genes and the highest variation in copy number among species. PKS;NRPSs and ChNPS12 subfamilies also have substantial variation in numbers of NRPS-encoding genes among species. **B**. Average and range (lowest to highest) of the number of A domains/NRPS in euascomycete genomes for each subfamily shows that the EAS subfamily also has by far the greatest variation in number of A domains/NRPS followed by the CYCLO, and SID subfamilies.

The range of variation in copy number of NRPS-encoding genes and in number of A domains/NRPS for each subfamily is shown for Euascomycete taxa only in Fig. 10. Variation in gene copy number is the highest for the EAS subfamily but both the PKS;NRPS and ChNPS12 subfamilies also show substantial variation (Fig. 10A). The EAS subfamily also shows by far the greatest variation in number of A domains/NRPS, followed by CYCLO and SID subfamilies, suggestive of less stable domain architectures and higher rates of intragenic domain duplication for these three groups. All of the remaining mono/bi-modular subfamilies show remarkably conserved domain architectures (Fig. 5, 10B), supporting available functional data which suggests these groups may have more central conserved roles in metabolism.

When we compared gene and domain duplication and loss in different subfamilies across euascomycetes, no particular subfamily showed significant evidence for nonrandom expansion or contraction of number of genes. When patterns of the total number of A domains per subfamily were analyzed, the EAS subfamily was the only group which showed highly significant (P < .00001) deviation from a random birth-death process (data not shown). These results support other observations that gain and loss of domains is an important evolutionary force within the EAS subfamily and may represent an adaptive response to niche-specific environmental pressures.

### Chain termination mechanisms

Our survey revealed that fungal NRPSs have a variety of C-terminal domains involved in chain termination. The most common for multimodular NRPSs is a C domain while for monomodular NRPSs it is an R domain (Additional file 2). R domains have previously been identified and shown to play a role in peptide release in fungal AARs [23,56], a number of fungal PKS;NRPSs [76-78], and in a minority of bacterial NRPSs including SafA and MxcG [20,21] and the PKS;NRPS hybrid, myxalamid [22]. Some multimodular NRPSs, however, also have a terminal R domain suggesting this may be a common release mechanism for fungal NRPSs. (Additional file 2). Two different release mechanisms have been identified for R type domains in fungal NRPSs, indicating the possibility of R domains subtypes. In fungal AAR's, the R domain reduces the enzyme bound α-aminoadipic acid [23]. The C-terminal R domain in the fungal PKS;NRPSs for Equisetin biosynthesis (EqiS), however, catalyzes a Dieckmann condensation reaction, thus performing a function similar to bacterial TE domains [76]. Some mono- and some multi-modular NRPSs terminate in T domains (Additional file 2) although these have not been implicated previously in chain release.

As noted previously, bacterial NRPSs generally have a TE domain at the C-terminal end for peptide release but TE domains have been found only in a few fungal NRPSs, notably the ACV synthetases [16]. We identified several other fungal NRPSs (AN2621.4, FGSG_11989.3, and Phchr1_2706), grouping with modules of Cyclosporin synthetase, which also contain a C-terminal TE domain (Additional file 2). However, our data suggest that TE domains are indeed rare in fungal NRPSs providing further support for the claims of horizontal transfer from bacteria to fungi of genes encoding ACV synthetases, and possibly these other fungal genes with TE domains.

## Conclusions

Phylogenomic analysis identified nine major subfamilies of fungal NRPSs which fall into two main groups: 1) a group of primarily mono/bi-modular enzymes (ChNPS10, AAR, ChNPS12, ChNPS11/ETP, PKS:NRPS, and CYCLO subfamilies) that group with bacterial NRPSs, and 2) a group of primarily multimodular proteins (EAS, SID) which appear both restricted to and highly expanded within fungi. Analyses demonstrate that α-aminoadipate reductases are more closely related to NRPSs than to other adenylating enzymes and provide further support for previous claims of horizontal transfer of certain NRPSs from bacteria to fungi. In addition, phylogenomic relationships among subfamilies, taxonomic distributions, structural conservation of domain architecture, and known data on function suggest that several of the mono/bi-modular groups are older in origin and play more central roles in cellular metabolism. The highly expanded group of fungal multimodular NRPSs, particularly the EAS subfamily, have less conserved domain architectures due to domain/module duplication and loss, and tend to perform more niche-specific functions, typically considered the realm of "secondary" metabolites.

## Methods

### Identification of putative NRPSs in fungal genomes

A set of fungal NRPSs with known chemical products was extracted from the NCBI database (Additional file 10), aligned using MUSCLE [102] with the 13 NRPSs identified previously in the Dothideomycete, *C. heterostrophus *C4 strain [10], and used to construct an initial HMMER model of fungal NRPS A domains using HMMER 2.0 http://hmmer.janelia.org (Additional file 11). This model was tested for specificity and ability to identify NRPSs proteins in fungal genomes for which NRPSs have been well characterized (e.g., *C. heterostrophus *and *Gibberella zeae/Fusarium graminearum*) and was found to correctly identify all known NRPSs in the genomes of these species as top hits. Protein datasets of a taxonomically representative sample of fungal genomes (Additional file 12) were downloaded and searched using both a local and global version of the fungal NRPS HMMER model. Proteins that were hit by our A domain model with an e-value less than 1 were considered possible NRPSs. A similar search strategy was employed on the nucleotide genome sequences using GENEWISE [103] and the same HMMER model to identify candidates that might have been missed or mis-annotated by automated gene calling programs. This approach did not identify any additional genes but did identify missed domains and also revealed a number of split gene annotations in the automated protein calls which we have reannotated. These included BC1G09040_09041.1, BC1G07441_07442.1, and FGSG11659.3 and FGSG11630.3 which we conclude represents a single gene corresponding to the MIPS and version 2 broad annotation (FG_00042.1), (Additional file 2).

For each fungal genome, A domains from all candidate NRPSs were aligned, using MUSCLE [102], with A domains from the 12 NRPSs previously identified from *C. heterostrophus *[10] (Additional file 1) and with A domains from related adenylating enzymes in the AMP-binding family (PFAM PF00501) [e.g., acyl CoA ligases (ACoAL), acetyl CoA synthetases (ACoAS), acyl AMP ligases (AAL), homologs of *C. heterostrophus *CPS1 (CPS1) [54], long chain fatty acid ligases (LCFAL), and homologs of Ochratoxin synthetase (OCHRA) [104] (Additional file 5). An initial phylogenetic analysis was conducted using the WAG+G model in PhyML to define a set of candidate NRPS proteins for each genome. Proteins from each genome grouping within a monophyletic group containing A domains of the known *C. heterostrophus *NRPS proteins and separated from the outgroup proteins with consistently high bootstrap support (>90), were retained in the dataset as candidate NRPSs or NRPS-like proteins. We chose to use individual A domains, rather than to include only proteins containing a complete A-T-C module as has been used in previous studies [105] because the latter would miss several putative NRPS or NRPS-like proteins (*e.g. C. heterostrophus *NPS10 and NPS12 [10]) that lack a complete A-T-C module. In addition, freestanding A domains in bacterial NRPSs have been shown to catalyze NRPS biosynthesis by activating and transferring substrates *in trans *to separate NRPSs [5] and the evolutionary relationship between monomodular NRPS-like proteins and multimodular NRPSs was also of interest.

### Annotation of domain architectures

All candidate proteins were annotated with our initial fungal NRPS A model and the PFAM models for C (PF00668) and T (PF00550) domains. Using the domains identified in the dataset from this search, a refined set of fungal specific NRPS HMMER models was built for the A (FungalNPSAMP.hmm), C (FungalNPSCON.hmm), and T (FungalNPSTHIOL.hmm) domains (Additional file 4). These models more accurately identified C and T domains in NRPSs with known/manually curated annotations than the generic PFAM models and were thus used to annotate A-T-C domain structures of all candidate fungal NRPSs. In addition, all candidate proteins were used as queries against the PFAM and INTERPRO domain databases to identify additional non-canonical NRPS domains present in these proteins. A complete domain architecture was compiled for each protein by merging these two approaches (Additional file 2).

### Phylogenomic analyses

Representatives of both fungal and bacterial adenylating enzymes used as outgroups (Additional file 5) in identification of putative NRPSs were also used as outgroups in phylogenomic analyses. While all AARs grouped as putative NRPSs, to reduce the size of the dataset, only a taxonomically representative sample of the fungal AARs were included in the full phylogenetic analyses. Fungal A domains from NRPSs with known function and/or chemical products present in GenBank were also included (Additional file 10). To select a diverse group of bacterial proteins, a representative A domain of each subfamily of fungal NRPSs was used to query the nr protein database at NCBI and the top 5 bacterial protein hits for each, as well as a number of bacterial proteins with known chemical products, were selected (Additional file 8). The complete set of A domains were extracted from these 58 bacterial proteins for a total of 99 A domains.

All candidate NRPS and outgroup A domains were aligned with MUSCLE [102]. Portions of ambiguous alignment were first adjusted manually and then masked to remove columns in the alignment with > 30% gaps prior to phylogenetic analysis (Additional file 13). A few candidate A domains were partial (BC1G15479, FG11319, AN8504, and Pa3740) and were removed from the final analysis because they did not align well with other NRPSs. ProtTest [106] was used to identify an appropriate protein substitution matrix as it has been shown that spurious choice of a matrix can lead to inaccurate phylogenies [107]. The RtREV+G+F model had the best likelihood score for all criteria (AIC and BIC) except for AIC-1 with sample size corrected for the number of sites in the alignment, which identified WAG+G as the best model. Three methods were used for phylogeny construction: 1) Maximum likelihood (ML) using RaxML [108] with the RtREV+G+F substitution model, 2) ML using PhyML with the WAG+G model [109], and 3) Neighbor joining (NJ) using NEIGHBOR in PHYLIP [110] and a distance matrix created in TREEPUZZLE [111] with the WAG+G substitution model. We used a Gamma distribution with four rate categories to model rate variation in all analyses. Bootstrapping was performed to assess the robustness of the phylogeny. Bootstrap datasets of 500 replicates for ML analysis and 200 replicates for the NJ analyses were created using SEQBOOT in PHYLIP and analyzed by the respective methods.

Because bootstrap support has been observed to decline in larger datasets [112-114], we also performed analyses on a subset of the data containing representatives from each of the major subfamilies identified. This dataset was aligned separately with MUSCLE and also masked with slightly less stringent conditions to remove columns containing greater than 50% gaps (Additional file 14). Phylogenetic analyses were performed on this dataset using the same methods described above.

Alignments have been deposited in TREEBASE (http://www.treebase.org/treebase/index.html, Study accession number = S2573 Matrix accession number = M4916).

### Subfamily identification and modelling

Fungal NRPS subfamilies were characterized as monophyletic groups defined by the most internal branch from the root above a bootstrap cutoff level (we chose 70%) [115,116] that also shared identical taxon composition across all three phylogenetic methods and had fungal NRPS representation (Additional file 6). The SID group was a single exception in that in the full phylogenies (Fig. 1, Additional file 6) maximum likelihood methods supported this clade with 68% and 74% bootstrap support while NJ did not provide support above 50% (Fig. 1, Additional file 6). This clade is, however, supported by >80% bootstrap support in all phylogenetic methods in analysis of the reduced dataset (Fig. 2, Additional file 7).

### Distribution of NRPS subfamilies across fungal taxonomic groups

To address patterns of distribution of NRPSs across fungal taxonomic groups, we tallied NRPS counts in Chytridiomycota, Zygomycota, Basidiomycota, Schizosaccharomycota, Hemiascomycota, and Euascomycota. Fisher's exact tests were used to test for associations between taxonomic groups and the proportion of genes in each NRPS subfamily.

### Lineage specific expansions and variation in birth-death rates

We calculated and graphed the average and range of the number of genes encoding NRPSs in each subfamily per euascomycete genome and the number of A domains per NRPS for each subfamily to assess broad patterns of variation in numbers of genes and numbers of A domains/gene across subfamilies (Fig. 10)

We used the method of Hahn et al. [117,118], which applies a stochastic birth and death process along a phylogeny to test for statistically significant lineage specific expansions and contractions of 1) number of NRPS genes and 2) numbers of NRPS A domains/subfamily. For these analyses, we created an ultrametric species tree with the PL method in r8s [119] using the phylogeny of the concatenated protein dataset of Fitzpatrick et al. [120] (Additional file 9).

We performed two separate analyses using CAFÉ [118] to look at patterns of gene and A domain expansions. The first analysis looked at patterns of the total number of NRPSs (e.g. all subfamilies combined) to look for broad patterns of expansions and contractions across the full tree of fungi (excluding *B. dendrobatidis*). The second analysis analyzed duplications and losses in each subfamily separately and was restricted to the euascomycete taxa because the birth-death model assumes that at least one gene of each subfamily is present in the common ancestor of all taxa. The ACV synthetase subfamily was excluded because parsimony inferred that this family had zero genes at the root. For all analyses, we used 1000 re-samplings and significant deviations from a random birth-death model were determined by viterbi p-values below .05.

All additional file figure legends and notes are in Additional file 15.

## Authors' contributions

BGT and KEB conceived of the study, and participated in its design, coordination and data interpretation. KEB carried out the protein and domain identifications, performed alignments, phylogenetic analyses, and drafted the manuscript. BGT advised KEB on manuscript content and in critical revisions. KEB and BGT jointly wrote the final versions of the manuscript. All authors read and approved the final manuscript.

## Supplementary Material

Additional file 1 **Diagram of *Cochliobolus heterostrophus *NRPSs and their domain structure**. 30 individual AMP domains are indicated. See Additional file 15 for detailed description. Click here for file

Additional file 2 **Annotation of all proteins used in the study**. Identification, accession numbers, genomic locations, and domain architectures of NRPSs identified in 38 fungal genomes. Click here for file

Additional file 3 **Genus and species abbreviations**. Genus and species abbreviations for all taxa used in the study. Click here for file

Additional file 4 **Profile HMMs for fungal-specific NRPS A, T, and C domains**. Zipped text file (file name extension .hmm to be used with the program package HMMER (http://hmmer.janelia.org). See Additional file 15 for detailed description. Click here for file

Additional file 5 **Fungal and bacterial AMP-binding protein outgroups**. Selection of fungal and bacterial AMP-binding protein used as outgroups in phylogenetic analyses. Click here for file

Additional file 6 **Phylogenies, full dataset**. **A**. NJ, **B**. ML (PhyML), and **C**. ML (RAxML) phylogenies of the full AMP dataset. See Additional file 15 for detailed description. Click here for file

Additional file 7 **Phylogenies, reduced dataset**. Topologies of NJ, ML (PhyML), and ML (RAxML) phylogenetic analyses of the reduced NRPS AMP domain dataset containing selected representatives of each subfamily. See Additional file 15 for detailed description. Click here for file

Additional file 8 **Bacterial outgroup proteins**. Bacterial proteins used as outgroups. Click here for file

Additional file 9 **Newick phylogenetic tree**. Text file containing newick phylogenetic tree for opening in tree visualization programs such as Treeview [121]. See Additional file 15 for detailed description. Click here for file

Additional file 10 **Known fungal NRPSs used for constructing the initial HMMER model**. Known fungal NRPS AMP domains used for constructing the initial HMMER model. Click here for file

Additional file 11 **HMMER AMP domain models used as the initial model for NRPS identification**. HMMER AMP domain models used as the initial model for NRPS identification. Zipped text files (file name extension .hmm to be used with the program package HMMER http://hmmer.janelia.org. Click here for file

Additional file 12 **Fungal protein datasets used in phylogenomic analyses**. Fungal protein (AMP domain) datasets used in phylogenomic analyses. Click here for file

Additional file 13 **MUSCLE alignment for complete AMP domain dataset**. MUSCLE alignment of 558 fungal and bacterial AMP domains used in phylogenetic analyses of the complete dataset. Zipped text file containing alignment in fasta format for visualization in sequence alignment editor such as ClustalX [122]. See Additional file 15 for detailed description. Click here for file

Additional file 14 **MUSCLE alignment for reduced AMP domain dataset**. MUSCLE alignment of the reduced dataset of fungal and bacterial AMP domains containing selected representatives of each major fungal subfamily and bacterial clades. Zipped text file containing alignment in fasta format for visualization in sequence alignment editor such as ClustalX [122]. See Additional file 15 for detailed description. Click here for file

Additional file 15 **Additional file figure legends and notes**. Selected additional file figure legends and notes. Click here for file
