# Supplementary material for: Phylogenomics reveals subfamilies of fungal nonribosomal peptide synthetases and their evolutionary relationships
Source: BMC Evol Biol. 2010 Jan 26;10:26. doi: 10.1186/1471-2148-10-26 (PMC2823734; doi:10.1186/1471-2148-10-26)
Supplement: Additional file 1 — Diagram of Cochliobolus heterostrophus NRPSs and their domain structure. 30 individual AMP domains are indicated. See Additional file 15 for detailed description. [file 1471-2148-10-26-S1.PDF]

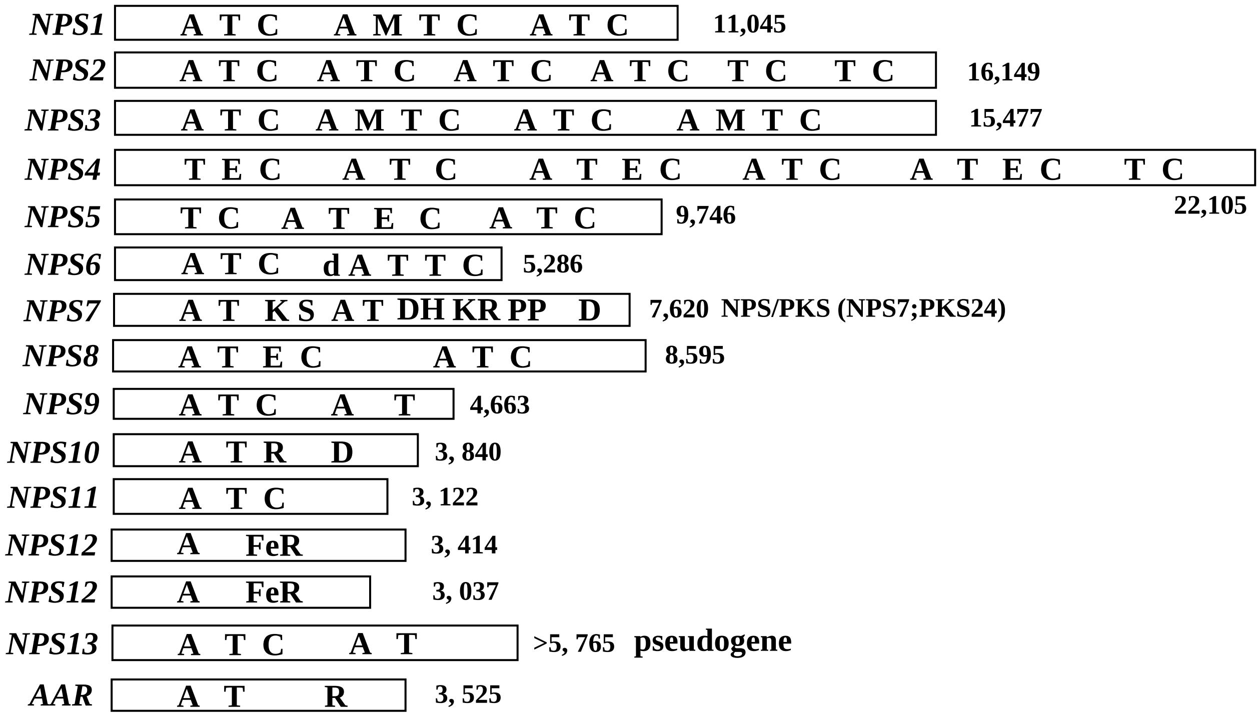

Additional File 1
