# Supplementary material for: Phylogenomics reveals subfamilies of fungal nonribosomal peptide synthetases and their evolutionary relationships
Source: BMC Evol Biol. 2010 Jan 26;10:26. doi: 10.1186/1471-2148-10-26 (PMC2823734; doi:10.1186/1471-2148-10-26)
Supplement: Additional file 2 — Annotation of all proteins used in the study. Identification, accession numbers, genomic locations, and domain architectures of NRPSs identified in 38 fungal genomes. [file 1471-2148-10-26-S2.PDF]

Additional File 2. Accession numbers, genome locations, and domain architectures of NRPSs identified in fungal genomes

Species	Sequencing Center/Version #	Sequence Center ID	Subfamily/ Group [a]	# AMP	Known Genes	Chromosomal Location	Domain Annotation [b]
Ashbya gossypii		ADL346W	AAR	1		ChmrIV: 96109:100266	A-T-R
Aspergillus fumigatus	CADRE/TIGR annotation	Afu1g10380	EAS	4	*Pes1*	Chromosome 1: 2675699-2694887 -	A-T-E-C-A-T-C-A-T-C-A-(DNALigA1)[c]-T-E-C-T-C-T
		Afu1g17200	SID	3	*SidC*	Chromosome 1: 4688800-4703141 +	A-T-C-A-T-C-A-T-C-T-C-T-C
		Afu3g15270	SIDE	2	*SidE*	Chromosome 3: 4010522-4017637 +	A-T-C-A-T-C
		Afu3g03350	SIDE	2	*SidE*	Chromosome 3: 891335-898767 +	A-T-C-A-T-C
		Afu3g03420	EAS	1	*NPS6*	Chromosome 3: 908168-914474 +	A-T-C-dA-T-C
		Afu3g12920	ETP	2		Chromosome 3: 3429981-3437235 +	A-T-C-A-T-C-T
		Afu3g13730	EAS	1		Chromosome 3: 3619321-3623193 +	A-T-C
		Afu4g14440	EAS	1		Chromosome 4: 3815691-3817758 -	A
		Afu4g11240	AAR	1		Chromosome 4: 2934763-2939218 +	A-T-R
		Afu5g12730	EAS	6		Chromosome 5: 3314537-3340084 +	A-T-C-A-T-E-C-A-T-C-T-E-C-A-T-C-A-T-C-A-T-E-C
		Afu5g10120	NPS10	1	*NPS10*	Chromosome 5: 2603086-2606910 +	A-T-R-D
		Afu6g09660	ETP	2	*GliP*	Chromosome 6: 2352620-2359124 -	A-T-C-A-T-C-T
		Afu6g09610	EAS	1		Chromosome 6: 2339538-2343356 -	A-T-C
		Afu6g12050	EAS	1		Chromosome 6: 3013593-3017507 +	A-T-C
		Afu6g12080	EAS	3		Chromosome 6: 3023316-3035305 -	A-T-C-A-T-E-C-A-T-C
		Afu6g03480	EAS	1		Chromosome 6: 748395-753511 +	A-M-T-R-(PLP)[c]
		Afu8g00170	EAS	2		Chromosome 8: 20854-27489 -	A-T-C-A-T-C
		Afu8g01220	NPS12	1		Chromosome 8: 286174-287750 +	A
		Afu8g00540	PKS;NPS	1		Chromosome 8: 117018-129323 +	KS-AT-M-KR-AC-C-A-T-R
		Afu8g01640	CYCLO	1		Chromosome 8: 430403-433447 -	A-T-R
Aspergillus nidulans	BROAD/Version 4	AN7884.4	EAS	6		Contig 43: 76779-97630 -	A-T-C-A-T-E-C-A-T-C-A-T-C-A-T-C-A-T-R

		AN2545.4	EAS	5		Contig 17: 152220-171385 +	T-E-C-A-T-E-C-A-T-C-A-T-C-A-T-C-A-T-C
		AN1242.4	EAS	4		Contig 2: 53533-71726 +	A-T-E-C-dA-C-A-T-C-A-(DNALigA1)[c]-T-E-C-T-C-T
		AN0016.4	EAS	4		Contig 45: 15391-26703 -	A-T-E-C-dA-A-T-C-A-T-E-C-T-C-T
		AN2621.4	ACV	3	*ACV*	Contig 7: 721978-736405 -	A-T-C-A-T-C-A-T-C-TE
		AN0607.4	SID	3	*SIDC*	Contig 59: 79653-86681 +	A-T-C-A-T-C-A-T-C-T-C-T-C
		AN3496.4	EAS	2		Contig 172: 18331-25266 -	T-C-A-T-C-A-T
		AN9244.4	EAS	2		Contig 107: 689-7055 +	A-T-C-T-C-(ESP)[c]-A
		AN6236.4	EAS	1	*NPS6*	Contig 59: 74210-78819 -	A-T-C-dA-T-C
		AN3495.4	EAS	1		Contig 79: 149178-154353 +	C-A-T-R
		AN10576.4	EAS	1		Contig 172: 14038-17366 -	A-T-E-C
		AN9243.4	EAS	1		Contig 153: 165693-167353 +	C-A
		AN8433.4	EAS	1		Contig 93: 164592-168404 +	A
		AN5318.4	NPS10	1	*NPS10*	Contig 153: 92194-104129 +	A-T-R-D
		AN8412.4	PKS;NPS	1		Contig 139: 270380-273613 -	KS-AT-M-KR-AC-C-A-T-R
		AN8105.4	CYCLO	1		Contig 153: 399039-401872 -	A-T-R
		AN8504.4[d]	incomplete			Contig 170: 179001-186848 -	dA-T-C
		AN9226.4	CYCLO/EAS	2		Contig 169: 212269-215635 +	A-T-C-A-M-T-C
		AN9129.4	NPS12	1		Contig 98: 7078-11515 +	A-FeR
		AN5610.4	AAR	1		Contig 98: 7078-11515 +	A-T-R
Batrachochytrium dendrobatidis	BROAD/Version 1	BDEG01579.1	AAR	1		Supercontig 1: 4224701-4229355 +	A-T-R
		BDEG03514.1	NPS12	1		Supercontig 4: 855028-860002 +	T-C-A-T-(FSH1)[c]
		BDEG08447.1	NPS12	1		Supercontig 16: 295121-301255 -	A-T-(RnaH)[c]-(LPS)[c]
Botrytis cinerea	BROAD/Version 1	BC1G10622.1	EAS	1		Supercontig 73: 102617-106612 -	A-T-C-R
		BC1G02495.1	EAS	2		Supercontig 8: 178697-187012 +	C-A-T-C-A-T-R

		BC1G10567.1	EAS	1	*NPS6*	Supercontig 72: 181976-187309 +	A-T-C-T-C
		BC1G03511.1	SID	3		Supercontig 13: 230111-245780 +	A-T-C-A-T-C-T-C-A-T-C-T-C-T-C
		BC1G10928.1	SID	3		Supercontig 75: 123029-134885 -	A-T-C-A-T-C-A-T-C-T-C
		BC1G04782.1	EAS	1		Supercontig 20: 111997-117883 -	M-T-C-A-(DNALigA1)[c]-T-R
		BC1G00695.1	PKS;NPS	1		Supercontig 2: 410037-422151 -	KS-AT-M-KR-AC-C-A-T-R
		BC1G15494.1	SID	1		Supercontig 180: 49908-52691 +	A-T-C
		BC1G09040_09041.1	EAS	3		Supercontig 52: 176500-186100 -	T-C-A-T-C-A-T-C-A-T-C-T
		BC1G15479.1 [d]	PKS;NPS	1		Supercontig 180: 3511-14382 -	KS-AT-M-KR-AC-C-A-T-R
		BC1G15703.1	PKS;NPS	1		Supercontig 196: 8963-16869 +	KS-AT-M-KR-AC-C-A-T-R
		BC1G07441_7442.1	ETP	1		Supercontig 42: 127734-130214 -	dA-T-C-T-C-A-T-C
		BC1G11613.1	other	1		Supercontig 91: 14,000-16,000	A-T-(Hx)[c]
		BC1G13197.1	AAR	1		Supercontig 116: 67595-71022 +	A-T-R
Candida albicans	BROAD/Version 1	CAWG_01102.1	AAR	1		Supercontig 1: 2601397-2605611 -	A-T-R
Candida glabrata	Genolevures/Version 1	CAGL0K07788g	AAR	1		Cagl0K: 774352-778476 -	A-T-R
Candida guilliermondii	BROAD/Version 1	PGUG_04759.1	AAR	1		Supercontig 6: 261996-266216 -	A-T-R
Candida lusitaniae	BROAD/Version	CLUG_04446.1	AAR	1		Supercontig 5: 712467-716633 +	A-T-R
Candida tropicalis		CTRG_04682.1	AAR	1		Supercontig 6: 899575-902463 +	A-T-R
Coccidioides immitis	BROAD/Version 3	CIMG09750.3	EAS	5		C. immitis RS: Chromosome 5: 2299495-2324157 -	A-T-E-C-A-T-C-A-T-T-C-C-A-C-A-T-C-T-C-T
		CIMG01429.3	EAS	1		C. immitis RS: Chromosome 1: 3743390-3749263 -	A-T-C-T-C
		CIMG03170.3	EAS	1		C. immitis RS: Chromosome 2: 630912-633926 -	C-A-T

		CIMG01861.3	EAS	2		C. immitis RS: Chromosome 1: 4899391-4906863 -	A-T-C-A-T-C
		CIMG07298.3	EAS	1		C. immitis RS: Chromosome 3: 4369404-4375473 -	A-T-C-T-C
		CIMG00941.3	SID	3		C. immitis RS: Chromosome 1: 2456614-2472250 -	A-T-C-A-T-C-T-C-A-T-C-T-C-T-C
		CIMG06629.3	PKS;NPS	1		C. immitis RS: Chromosome 3: 2485975-2498131 -	KS-AT-M-KR-AC-C-A-T-R
		CIMG01491.3	AAR	1		C. immitis RS: Chromosome 1: 3907134-3911528 -	A-T-R
Cochliobolus heterostrophus	JGI/Version 1	CocheC5_1_29312	NPS10	1	*NPS10*	CocheC5_1/ scaffold_6: 1384455-1390709	A-T-R-D
		CocheC5_1_115564	ChNPS11/ ETPm1	1	*NPS11*	CocheC5_1/scaffold _1:1126440-1130558	A-T-C
		CocheC5_1_118012	NPS12	1	*NPS12*	CocheC5_1/scaffold _11:763264-766881	A-FeR
		CocheC5_1_116719	NPS12	1	*NPS12*	CocheC5_1/scaffold _5:262551-266118	A-FeR
		CocheC5_1_15959	2CYCLO/ 2EAS	4	*NPS3*	CocheC5_1/scaffold _1:554870-569203	A-T-C-A-M-T-C-A-T-C-A-M-T-C
		CocheC5_1_84777	1CYCLO/ 2EAS	3	*NPS1*	CocheC5_1/scaffold _6:788062-801107	A-T-C-A-M-T-C-A-T-C
		CocheC5_1_115936	AAR	1	*AAR1*	CocheC5_1/scaffold _2:839177-843985	A-T-R
		CocheC5 _1_77609	SID	4	*NPS2*	CocheC5_1/scaffold _33:136682-152804	A-T-C-A-T-C-A-T-C-A-T-C-T-C-T-C
		CocheC5_1_16574	EAS	1	*NPS13*	CocheC5_1/ scaffold_1: 2669103-2670314	A-T
		CocheC5_1_3317	EAS	1	*NPS6*	CocheC5_1/ scaffold_ 25: 568366-575312	A-T-C-dA-T-T-C
		CocheC5_1_94644	EAS	2	*NPS5*	CocheC5_1/scaffold _25:25248-35993	T-C-A-T-E-C-A-T-C
		CocheC5_1_94248	EAS	2	*NPS8*	CocheC5_1/scaffold _23:520703-531586	A-T-E-C-A-T-C
		CocheC5_1_119280	EAS	2	*NPS9*	CocheC5_1/scaffold _23:2629-8556	A-T-C-A-T
		CocheC5_1_112395	EAS	4	*NPS4*	CocheC5_1/scaffold _22:508445-531549	T-E-C-A-T-C-A-T-E-C-A-T-C-A-T-E-C-T-C
		CocheC5_1_89648	MBC	1	*NPS7*	CocheC5_1/scaffold _13:211976-223348	A-T-KS-AT-DH-KR-T-D

Coprinus cinereus	BROAD/Version 2	CC1G_03009.2	NPS12	1		Contig 177: 344918-348700 -	A-FeR
		CC1G_04210.2	SID	1		Contig 105: 74273-82054 -	A-T-C-T-C-T-C
		CC1G_06235.2	NPS12	1		Contig 194: 186935-190731 -	A-FeR
		CC1G_06250.2	NPS12	1		Contig 194: 233847-237602 -	A-FeR
		CC1G_15694.2	AAR	4		Contig 11: 933268-937996 -	A-T-R
Cryptococcus neoformans	BROAD/Version 1	CNAG_03588.1	AAR	1		Chromosome 8: 1345985-1350502 -	A-T-R
Debaromyces hansenii	Genolevures/Version 1	DEHA2D07964g	AAR	1		Deha2D – 684912-653108	A-T-R
Encephalitozoon cuniculi	NCBI/unannotated	none					
Fusarium graminearum	BROAD/Version 3	FGSG_11659.3 [e]	EAS	7	*NPS8*	F. graminearum: Supercontig 1: 144805-158252 -	A-T-C-A-T-C-A-T-C-A-T-C-A-T-C-A-T-C-A-T-C
		FGSG_11660.3 [e]			*NPS8*	F. graminearum: Supercontig 1: 162481-165335 -	
		FGSG_13783.3	EAS	6	*NPS18*	F. graminearum: Supercontig 7: 2213860-2242925 +	A-T-C-A-T-E-C-A-T-E-C-A-T-E-C-A-T-E-C-A-T-C
		FGSG_02315.3	EAS	5	*NPS4*	F. graminearum: Supercontig 1: 7439120-7462127 -	A-T-E-C-A-T-C-A-T-E-C-A-T-C-A-T-E-C-T-C
		FGSG_02394.3	EAS	2	*NPS15*	F. graminearum: Supercontig 1: 7670431-7677408 -	A-T-C-A-T-R
		FGSG_08209.3	EAS	3	*NPS7*	F. graminearum: Supercontig 5: 2569253-2582878 -	T-C-A-T-C-A-T-C-A-T-C
		FGSG_05372.3	SID	3	*NPS2*	F. graminearum: Supercontig 3: 2043338-2057970 +	A-T-C-A-T-C-T-C-A-T-C-T-C-T-C
		FGSG_11026.3	SID	3	*NPS1*	F. graminearum: Supercontig 8: 574013-588387 +	A-T-C-A-T-C-A-T-C-T-C-T-C
		FGSG_11395.3	EAS	2	*NPS14*	F. graminearum: Supercontig 9: 312651-319899 +	A-T-C-A-T-C
		FGSG_03747.3	EAS	1	*NPS6*	F. graminearum: Supercontig 2: 2809478-2815749 -	A-T-C-T-C

		FGSG_01680.3	EAS	1	*NPS16*	F. graminearum: Supercontig 1: 5534719-5539722 -	A-M-T-R-(PLP)[c]
		FGSG_13878.3	EAS	8	*NPS5*	F. graminearum: Supercontig 8: 693139-727216 +	A-C-A-T-E-C-A-T-E-C-A-T-E-C-A-T-E-C-A-T-E-C-A-T-E-C-A-T-R
		FGSG_10990.3	EAS	1	*NPS9*	F. graminearum: Supercontig 8: 686748-689261 -	A-T
		FGSG_10523.3	EAS	1	*NPS3*	F. graminearum: Supercontig 7: 2137744-2145297 -	T-E-C-A-T-C-T
		FGSG_10702.3	EAS	1	*NPS17*	F. graminearum: Supercontig 7: 2680651-2682687 -	A
		FGSG_11294.3	NPS12	1	*NPS12*	F. graminearum: Supercontig 9: 577120-580295 +	A-FeR
		FGSG_06507.3	NPS10	1	*NPS10*	F. graminearum: Supercontig 4: 265804-269866 -	A-T-R-D
		FGSG_03245.3	NPS12	1	*NPS11*	F. graminearum: Supercontig 2: 4162913-4166150 -	A-FeR
		FGSG_11989.3	CYCLO	1	*NPS19*	F. graminearum: Supercontig 1: 5556950-5560682 +	A-M-T-TE
		FGSG_13153.3	NPS12	1	*NPS13*	F. graminearum: Supercontig 4: 3627883-3631142 -	A-FeR
		FGSG_06041.3	AAR	1		F. graminearum: Supercontig 3: 4113978-4117801 +	A-T-R
		FGSG_07798.3	PKS;NPS	1		F. graminearum: Supercontig 4: 4503929-4515787 -	KS-AT-M-KR-AC-C-A-T-R
		FGSG_11319.3[d]	incomplete			F. graminearum: Supercontig 9: 520033-520572 -	A
Kluveromyces lactis var. *lactis*	Genolevures/Version 1	KLLA0B09218g	AAR	1		KllaOB 805915-810072	A-T-R
Laccaria bicolor	JGI/Version 1	Lacbi1_150981	AAR	1		scaffold_9: 88330-92826	A-T-R
Magnaporthe oryzae	BROAD/Version 6	MGG_07858.6	EAS	4		Supercontig 183: 547109-561691	A-T-C-A-T-C-A-T-C-A-T-C
		MGG_02351.6	EAS	5		Supercontig 186: 3232658-3248975 -	A-T-C-A-T-C-A-T-C-A-T-C-A-T-C

		MGG_00022.6	2CYCLO/1 EAS	3		Supercontig 194: 4090279-4102482 +	A-T-C-A-M-T-C-A-T-C
		MGG_09589.6	PKS;NPS	1		Supercontig 197: 596671-608484 -	KS-AT-M-KR-AC-C-A-T
		MGG_03290.6	NPS10	1	*NPS10*	Supercontig 190: 371071-374913 +	A-T-R-D
		MGG_07803.6	ChNPS11/ ETPm1	1		Supercontig 183: 323819-328757 +	C-A-T-KS
		MGG_15248.6	ChNPS11/ ETPm1	1		Supercontig 183: 125103-133264 +	T-C-A-T-C
		MGG_03401.6	EAS	1		Supercontig 190: 3214-11859 -	T-E-C-A-T-C
		MGG_14943.6	PKS;NPS	1		Supercontig 187: 767479-771399 -	KS-AT-M-KR-AC-C-A-T-R
		MGG_14897.6	PKS;NPS	1	*SYN8*	Supercontig 187: 2269449-2280593 -	KS-AT-M-KR-AC-C-A-T-R
		MGG_03810.6	PKS;NPS	1		Supercontig 187: 735169-748461 -	KS-AT-M-KR-AC-C-A-T-R
		MGG_12175.6	SID	3	*NPS2*	Supercontig 187: 2165442-2180544 -	A-T-C-A-T-C-T-C-A-T-C-T-C-T-C
		MGG_14967.6	other	4		Supercontig 187: 2952752-3007190 +	A-T-C-T-C-A-T-C-T-C-A-T-E-C-A-T-C
		MGG_04949.6 [d]	incomplete			Supercontig 21: 78-3628 -	A
		MGG_12447.6	PKS;NPS	1	*SYN2*	Supercontig 195: 2333033-2345385 -	KS-AT-M-KR-AC-C-A-T-R
		MGG_15097.6	PKS;NPS	1	*ACE1*	Supercontig 195: 2390311-2400256 +	KS-AT-M-KR-AC-C-A-T-R
		MGG_11222.6	NPS12	1	*NPS12*	Supercontig 196: 1938606-1939504 -	A-FeR
		MGG_14767.6	EAS	1	*NPS6*	Supercontig 196: 2997845-2999481, +Supercontig 196: 2999838-3004145 +	A-T-C-dA-T-C
		MGG_02611.6	AAR	1		Supercontig 193: 2144939-2148616 +	A-T-R
Neurospora crassa	BROAD/Version 3	NCU07119.3	SID	3		Contig 34: 78380-93958 -	A-T-C-A-T-C-T-C-A-T-C-T-C-T-C
		NCU08441.3	EAS	1	*NPS6*	Contig 44: 204858-211015 +	A-T-C-dA-T-C
		NCU04531.3	EAS	1		Contig 21: 527341-536094 -	T-T-C-C-A-T-C
		NCU03010.3	AAR	1		Contig 7: 160134-163731 -	A-T-R
Phanaerochaete chrysosporium	JGI/Version 1	Phchr1_2706	CYCLO	2		scaffold_11: 866634-867772	A-M-T-C-A-T-TE
		Phchr1_135156	NPS12	1		scaffold_20: 228019-231930	A-FeR

		Phchr1_161268	AAR	1		scaffold_2: 1745018-1748359	A-T-R
Phycomyces blakesleeanus	JGI/Version 1	Phybl1_34455	AAR	1		Phybl1/scaffold_53: 8827-13373	A-T-R
Pichia stipitis	JGI/Version 2	Picst3_68020	AAR	1		Picst3/chr_6.1:353493-357715	A-T-R
Podospora anserina	Genoscope/Version 1	Pa0_240	PKS;NPS	1		SC_C_chrm6_seq: 84078..97458	KS-AT-M-KR-AC-C-A-T-R
		Pa1_5210	PKS;NPS	1		SC_D_chrm1_seq: 335192..336221	KS-AT-M-KR-AC-C-A-T-R
		Pa2_7870	SIDE	2		SC_B_chrm2_seq: 4277923..4279120	C-A-T-C-A-T-C
		Pa3_11200	EAS	1	*NPS6*	SC_C_chrm3.seq: 250296..255914	A-T-C-T-C
		Pa4_4440	SID	3		SC_D_chrm4.seq: 93610..108867	A-T-C-A-T-C-T-C-A-T-C-T-C-T-C
		Pa4_4630	EAS	4		SC_D_chrm4.seq: 167899..184030	A-T-E-C-A-T-C-A-T-E-C-A-(DNALigA1)[c]-T
		Pa4_4640	EAS	1		SC_D_chrm4.seq: 184183..192897	A-T-E-C-A-T-C-A-T-E-C-A-T-C-A-T-E-C-T-C-T
		Pa5_1070	EAS	1		SC_A_chrm5.seq: 416202..424777	T-T-(PI4S)[c]-C-C-A-T-C
		Pa5_6830	PKS;NPS	1		SC_E_chrm5.seq: 175989..188303	KS-AT-M-KR-AC-C-A-T-R
		Pa5_3740 [d]	incomplete	I		SC_B_chrm5.seq: 206605..208782	C-dA
		Pa6_10100	PKS;NPS	1		SC_D_chrm6_seq:622720..628038-	C-A-T-T-R
		Pa0_670	NPS12	1		SC_A_chrm6.seq: 27621..30710	A-T-C
		Pa1_5110	AAR	1		SC_B_chrm1.seq: 1478700..1482367	A-T-R
Postia placenta	JGI/Version 1	Pospl1 111174	NPS12	1		Pospl1/scaffold_133:154647-156802	A
		Pospl1 95457	NPS12	1		Pospl1/scaffold_133:61362-63334	A
		Pospl1 42387	NPS12	1		Pospl1/scaffold_140:198705-199322	A
		Pospl1 127321	NPS12	1		Pospl1/scaffold_133:159978-162012	A
		Pospl1 49678	NPS12	1		Pospl1/scaffold_133:50945-52834	A
		Pospl1 54576	NPS12	1		Pospl1/scaffold_34: 368246-370135	A

		Pospl1 42034	NPS12	1		Pospl1/scaffold_43: 296234-296941	A
		Pospl1 54642	NPS12	1		Pospl1/scaffold_34: 361746-362573	A
		Pospl1_109769	AAR	1		Pospl1/scaffold_11: 161253-165967	A-T-R
		Pospl1_115736	AAR	1		Pospl1/scaffold_12: 340539-344819	A-T-R
Puccinia graminis	BROAD/Version 2	PGTG_06519.2	other	1		Supercontig 15: 236832-241982 -	A-T-C
		PGTG_07683.2	AAR	1		Supercontig 20: 1032995-1037380+	A-T-R
Rhizopus oryzae	BROAD/Version 1	RO3G_12433.1	AAR	1		Supercontig 10: 1228709-1233004+	A-T-R
Saccharomyces cerevisiae	SGD,BROAD/Version 1	YBR115C/ SCRG_02851.1	AAR	1		Chr2: 473920-469742 -	A-T-R
Saccharomyces bayanus	BROAD/unannotated	none	AAR	1		contig_7_ 37512-38720	A-T-R
Saccharomyces mikatae	BROAD/unannotated	none	AAR	1		contig_91_ 4043-5791	A-T-R
Saccharomyces paradoxus	BROAD/unannotated	none	AAR	1		contig_203_ 6588-4838	A-T-R
Schizosaccharo-myces japonicus	BROAD/Version 2	SJAG_04031.2	SID	3	*sib1*	S. japonicus yFS275: Supercontig 5: 577316-591914 -	A-T-C-T-C-T-C-A-T-C-T-C-T-C
		SJAG_00869.2	AAR	1		Supercontig 1: 1769595-1773839+	A-T-R
Schizosaccharo-myces pombe	BROAD/Version 2	SPAP7G5.04c	AAR	1	*LYS2*	Chromosome 1: 3739162-3743421 -	A-T-R
		SPAC23G3.02c	SID	3	*sib1*	Chromosome 1: 854523-869527 -	A-T-C-T-C-T-A-T-T-C-T-C
Sporobolomyces roseus	JGI/Version 1	Sporo1_21452	AAR	1		Sporo1/scaffold_3:1 748229-1753072	A-T-R
		Sporo1_31423	other	1		Sporo1/scaffold_1:2 360477-2373012	A-T-C-T
Trichoderma reesii	JGI/Version 2	Trire2_123786	EAS	14		Trire2/scaffold_26:2 76834-327620	KS-AT-T-C-A-T-C-A-T-C-A-T-C-A-T-C-A-T-C-A-T-C-A-T-C-A-T-C-A-T-C-A-T-C-A-T-C-A-T-C-A-T-C-A-T-R

		Trire2_23171	EAS	20	*TEX1* homolog	Trire2/scaffold_24:123560-193077	
		Trire2_58285	PKS;NPS	1		Trire2/scaffold_5:25618-37773	KS-AT-M-KR-AC-C-A-T-R
		Trire2_59315	PKS;NPS	1		Trire2/scaffold_6:34746-46569	KS-AT-M-KR-AC-C-A-T-R
		Trire2_60751	EAS	1		Trire2/scaffold_8:524121-526840	A-T-C
		Trire2_67189	EAS	1	*NPS6*	Trire2/scaffold_20:536612-542053	A-T-C-T-C
		Trire2_68204	NPS12	1		Trire2/scaffold_24:269353-272919	A-FeR
		Trire2_71005	EAS	1	*NPS6*	Trire2/scaffold_1:3561799-3563725	A-T-C-T-C
		Trire2_81014	NPS10	1	*NPS10*	Trire2/scaffold_22:47424-51332	A-T-R-D
		Trire2_24586	ChNPS11/ETPm1	2		Trire2/scaffold_1:2715208-2721935	A-T-C-A-T-C-T
		Trire2_60458	ChNPS11/ETPm1	2		Trire2/scaffold_7:1346092-1352757	A-T-C-A-T-C-T
		Trire2_69946	SID	3		Trire2/scaffold_31:39879-54649	A-T-C-A-T-C-T-C-A-T-C-T-C-T-C
		Trire2_4117	AAR	1		Trire2/scaffold_10:721901-725674	A-T-R
Ustilago maydis	BROAD/Version 1	UM05165.1	SID	3	*sid2*	Contig 188: 245412-257254 +	A-T-C-A-T-C-A-T-C-T-C
		UM01434.1	SID	3	*fer3*	Contig 49: 92548-107141 +	A-T-C-A-T-C-A-T-C-T-C-T-C
		UM05245.1	other	3		Contig 191: 1-10972 -	A-T-C-A-T-C-A-T
		UM03108.1	NPS10	1	*NPS10*	Contig 105: 12433-16395 +	A-T-R-D
		UM01697.1	AAR	1		Contig 66: 37971-42527 +	A-T-R
Yarrowia lipolytica	Genolevures/Version 1	YALI0E06457g	AAR	1		Yali0E: 734132..738373 +	A-T-R

[a] See Table 1.

[b] Domain codes:

	Interpro #	PFAM	
A	IPR000873	PF00501	AMP-dependent synthetase and ligase
T	IPR006162	PF00550	Phosphopantetheine attachment site
C	IPR001242	PF00668	Condensation
E	IPR001509	PF01370	Epimerization
M	IPR013217	PF08242	Methyltransferase type 11 and type 12
R	IPR010080		Thioester reductase
D	IPR002198	PF00106	Short-chain dehydrogenase/reductase
KS	IPR014030	PF00109	Beta-ketosynthase

KR	IPR013968	PF08659	Keto-reductase
AT	IPR014043	PF00698	Acyl transferase
AC	IPR009081		Acyl carrier protein-like
TE	IPR001031	PF00975	Thioesterase
FeR	IPR013130	PF01794	Ferric reductase transmembrane domain
3BHS	IPR002225	PF01073	3-Beta hydroxysteroid dehydrogenase/isomerase
N4	IPR013120	PF07993	NAD_binding_4-male sterility factor
HX	IPR001451	PF00132	Bacterial transferase hexapeptide repeat
FSH1	IPR006660	PF03960	FSH1 - Serine Hydolase
PLP	IPR018319	PF03841	Pyridoxal phosphate-dependent transferase
LPS	IPR006629		LPS-induced tumor necrosis factor alpha factor
RnaH	IPR012337		Polynucleotidyl transferase, Ribonuclease H fold
ESP	IPR001638		Extracellular solute-binding protein, family 3
FabD	IPR016035		FabD/lysophospholipase-like
PI4S	IPR000215		Protease inhibitor I4, serpin
DNAligA1	IPR016059		DNA Ligase A1

[c] Domains in parentheses indicate domains which are noncannonical or unusual NRPS domains with hits less than e-10

[d] These NRPSs were removed from the final phylogenetic analyses as only a partial A domain, that did not align well with other sequences, was identified.

[e] Our annotation of genomic DNA suggests that these two genes (FGSG_11659.3 and FGSG_11660.3) should be merged to form a single gene with seven A-T-C modules that corresponds to the FG00042.1 in BROAD annotation of *F. graminearum,* version 1. The gene is referred to as FG00042.1 in all trees, figures, and tables.
