# Supplementary material for: Phylogenomics reveals subfamilies of fungal nonribosomal peptide synthetases and their evolutionary relationships
Source: BMC Evol Biol. 2010 Jan 26;10:26. doi: 10.1186/1471-2148-10-26 (PMC2823734; doi:10.1186/1471-2148-10-26)
Supplement: Additional file 3 — Genus and species abbreviations. Genus and species abbreviations for all taxa used in the study. [file 1471-2148-10-26-S3.PDF]

Additional File 3. Genus and species abbreviations

Fungi

Acremonium chrysogenum	*Ac*
Alternaria alternata	*Aa*
Alternaria brassicae	*Ab*
Ashbya gossypii	*Ag*
Aspergillus fumigatus	*Af*
Aspergillus nidulans	*An*
Batrachochytrium dendrobatidis	*Bd*
Botrytis cinerea	*Bc*
Candida albicans	*Ca*
Candida glabrata	*Cgl*
Candida guilliermondii	*Cgu*
Candida lusitaniae	*Cl*
Candida tropicalis	*Ct*
Claviceps purpurea	*Cp*
Coccidioides immitis	*Ci*
Cochliobolus carbonum	*Cca*
Cochliobolus heterostrophus	*Ch*
Coprinus cinereus	*Cc*
Cryptococcus neoformans	*Cn*
Debaryomyces hansenii	*Dh*
Encephalitozoon cuniculi	*Ecu*
Epichloë festuca	*Ef*
Fusarium equiseti	*Fe*
Fusarium graminearum	*Fg*
Fusarium heterosporum	*Fh*
Gibberella fujikuroi	*Gf*
Hypocrea virens	*Hv*
Kluyveromyces lactis	*Kl*
Laccaria bicolor	*Lb*
Leptosphaeria maculans	*Lm*
Magnaporthe oryzae	*Mg*
Metarhizium anisopliae	*Ma*
Neurospora crassa	*Nc*
Penicillium chrysogenum	*Pc*
Phanaerochaete chrysosporium	*Pch*
Phycomyces blakesleeanus	*Pb*
Pichia stipitis	*Ps*
Podospora anserina	*Pa*
Postia placenta	*Pp*
Puccinia graminis	*Pg*
Pyrenophora tritici-repentis	*Pt*
Rhizopus oryzae	*Ro*
Saccharomyces bayanus	*Sb*
Saccharomyces cerevisiae	*Sc*
Saccharomyces mikatae	*Sm*
Saccharomyces paradoxus	*Spa*
Schizosaccharomyces japonicus	*Sj*
Schizosaccharomyces pombe	*Sp*
Sporobolomyces roseus	*Sr*
Trichoderma reesii	*Tr*
Tolypocladium inflatum	*Ti*
Ustilago maydis	*Um*
Yarrowia lipolytica	*Yl*

Bacteria

Anabaena variabilis	*Av*
Arthrobacter sp.	*Asp.*
Bacillus amyloliquefaciens	*Ba*
Bacillus subtilis	*Bs*
Brevibacillus brevis	*Bb*
Brevibacillus parabrevis	*Bp*
Brevibacillus texasporus	*Bt*
Burkholderia cenocepacia	*Bce*
Chlorobium ferrooxidans	*Cf*
Clostridium cellulolyticum	*Cce*
Crocosphaera watsonii	*Cw*
Cyanothece sp.	*Csp.*
Dinoroseobacter shibae	*Ds*
Escherichia coli	*Ec*
Geobacter sulfurreducens	*Gs*
Hahella chejuensis	*Hc*
Herpetosiphon aurantiacus	*Ha*
Heliobacterium modesticaldum	*Hm*
Lyngbya majuscule	*Lma*
Lysobacter lactamgenus	*Ll*
Melittangium lichenicola	*Ml*
Microcystis aeruginosa	*Mae*
Micromonospora sp.	*Msp.*
Mycobacterium tuberculosis	*Mt*
Myxococcus xanthus	*Mx*
Nocardia lactamdurans	*Nl*
Nodularia spumigena	*Ns*
Nostoc punctiforme	*Np*
Nostoc sp.	*Nsp.*
Opitutus terrae	*Ot*
Photorhabdus luminescens	*Pl*
Pseudomonas aeruginosa	*Pae*
Pseudomonas entomophila	*Pe*
Pseudomonas fluorescens	*Pf*
Pseudomonas putida	*Ppu*
Pseudomonas syringae	*Psy*
Rhodococcus jostii	*Rj*
Roseobacter denitrificans	*Rd*
Salinispora arenicola	*Sa*
Salinispora tropica	*St*
Shewanella oneidensis	*So*
Stigmatella aurantiaca	*Sa*
Streptomyces clavuligerus	*Scl*
Streptomyces coelicolor	*Sco*
Yersinia pestis	*Yp*
