# Supplementary material for: Phylogenomics reveals subfamilies of fungal nonribosomal peptide synthetases and their evolutionary relationships
Source: BMC Evol Biol. 2010 Jan 26;10:26. doi: 10.1186/1471-2148-10-26 (PMC2823734; doi:10.1186/1471-2148-10-26)
Supplement: Additional file 5 — Fungal and bacterial AMP-binding protein outgroups. Selection of fungal and bacterial AMP-binding protein used as outgroups in phylogenetic analyses. [file 1471-2148-10-26-S5.PDF]

Additional File 5. Fungal and bacterial AMP-binding protein outgroups

Species	NCBI Accession	Genome Sequencing Center ID	Protein
**α-amino-adipate reductases**			
Aspergillus fumigatus	XP_751705.1	Afu4g11240	
Rhizopus oryzae	XP_001879618.1	RO3G12433.1	
Batrachochytrium dendrobatidis	XP_001879618.1	BDEG_1579.1	
Cochliobolus heterostrophus		CocheC5_115936	
Debaryomyces hansenii	XP_001385417.1	DEHA0D08734g	
Fusarium graminearum	XP_386217.1	FGSG06041.3	
Schizosaccharomyces pombe	CAB88271.1	SPAP7G5.04c	Lys1
Saccharomyces cerevisiae	NP_009673.1	YBR115C/ SCRG_02851.1	Lys2
Neurospora crassa	XP_965396.1	NCU03010.3	
Phycomyces blakesleeanus	XP_001879618.1	Phybl1_34455	
Ustilago maydis	XP_757844.1	UM01697.1	
**4-Coumarate/Acyl-CoA Ligases**			
Cochliobolus heterostrophus		CocheC5_97601	
Fusarium graminearum	XP_383765.1	FGSG03589.3	
Rhizopus oryzae		RO3G05716.3	
Ustilago maydis	XP_757318.1	UM01171.1	
Alternaria alternata	BAB6907.1		Aft
Alternaria alternata	BAA36588.1		Akt1
Mycobacterium tuberculosis	YP_001135507.1	Mflv_4250	
Arthrobacter sp.	YP_833499.1	Arth_4024	
Streptomyces coelicolor	NP_628552.1	SCO4383	
Dinoroseobacter shibae	YP_001531603.1	Dshi_0253	
Roseobacter denitrificans	YP_682165.1	RD1_1868	
Arthrobacter sp.	YP 833499.1	Arth_4024	
Streptomyces coelicolor	NP 624638.1	SC5G9.20	
Rhodococcus jostii	YP_705267.1	RHA1_ro05328	
**Acetyl CoA Synthetases**			
Aspergillus fumigatus	XP_751720.1	Afu4g11080	
Batrachochytrium dendrobatidis		BDEG00471.1	
Cochliobolus heterostrophus		CocheC5_11359	
Saccharomyces cerevisiae	EDV09449.1	SCRG_05132.2	
Schizosaccharomyces pombe	NP_588291.1	SPCC417.14c	
Ustilago maydis	XP_759216.1	UM_03069.1	
Escherichia coli	NP_756916.1	c5064	
Yersinia pestis	NP_403903.1	YPO0253	
Pseudomonas syringae	NP_791649.1	PSPTO_1825	
Shewanella oneidensis	NP_718327.1	SO_2743	
**Acyl AMP Ligases (AALs)**			
Aspergillus fumigatus	XP_752870.1	Afu1g15010	Cps1
Cochliobolus heterostrophus	AAG53991.2	CocheC5_66090	Cps1
Schizosaccharomyces pombe	NP_593217.1	SPAC56F8.02	Cps1
Fusarium graminearum	AAP12366.1	FGSG_06631.3	Cps1
Saccharomyces cerevisiae	EDV10692.1	Y0R093C/SCRG_01491.2	Cps1
Myxococcus xanthus	AAC44128.1	U24657.1	SafB
Mycobacterium tuberculosis	YP_001284310.1	MRA_2967	FadD28
Lyngbya majuscule	AAS98774.1		JamA
Bacillus subtilis	AAF08795.1		MycA
Stigmatella aurantiaca	ZP_01464049.1	STIAU_1156	

**Long Chain Fatty Acid Acyl CoA Ligases (LCFAL)**		
Cochliobolus heterostrophus		CocheC5_31926
Ustilago maydis	XP_760950.1	UM04803.1
Aspergillus fumigatus	XP_753087.1	Afu1g17190
Neurospora crassa	XP_965748.1	NCU00608.3
Mycobacterium tuberculosis	NP_217021.1	Rv2505c
Geobacter sulfurreducens	NP_952156.1	GSU1103
Burkholderia cenocepacia	YP_002092711.1	BCPG_01457.1
Heliobacterium modesticaldum	YP_001678729.1	HM1_0093
**Ochratoxin (OCHRA)**		
Aspergillus fumigatus	XP 748589.1	Afu3g02670
Pyrenophora tritici-repentis	XP_001936483.1	PTRG_06150.2
Neurospora crassa	XP 955820.1	NCU05000.3
Fusarium graminearum	XP_390793.1	FGSG_10617.3
Botrytis cinerea	XP 001558652.1	BC1G_02723.1

Blank = none or not known
