# Supplementary figures and images for: Phylogenomics reveals subfamilies of fungal nonribosomal peptide synthetases and their evolutionary relationships
Source: BMC Evol Biol. 2010 Jan 26;10:26. doi: 10.1186/1471-2148-10-26 (PMC2823734; doi:10.1186/1471-2148-10-26)

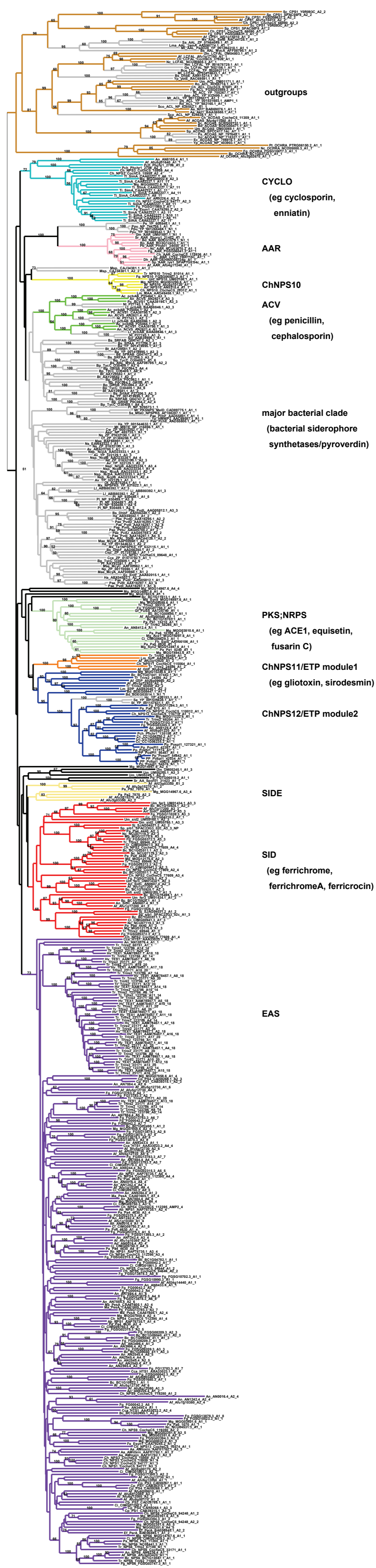

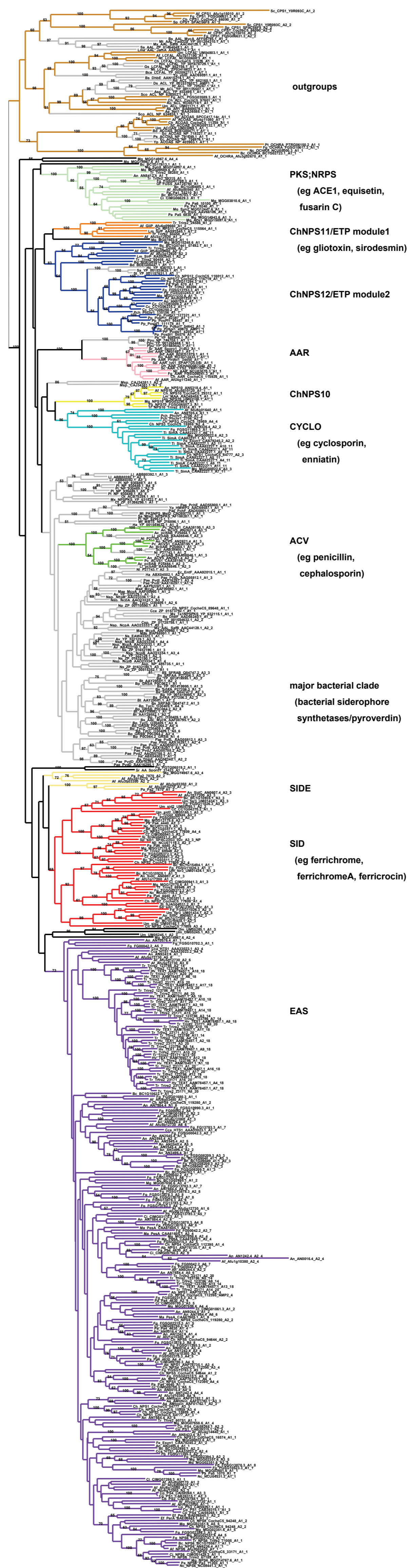

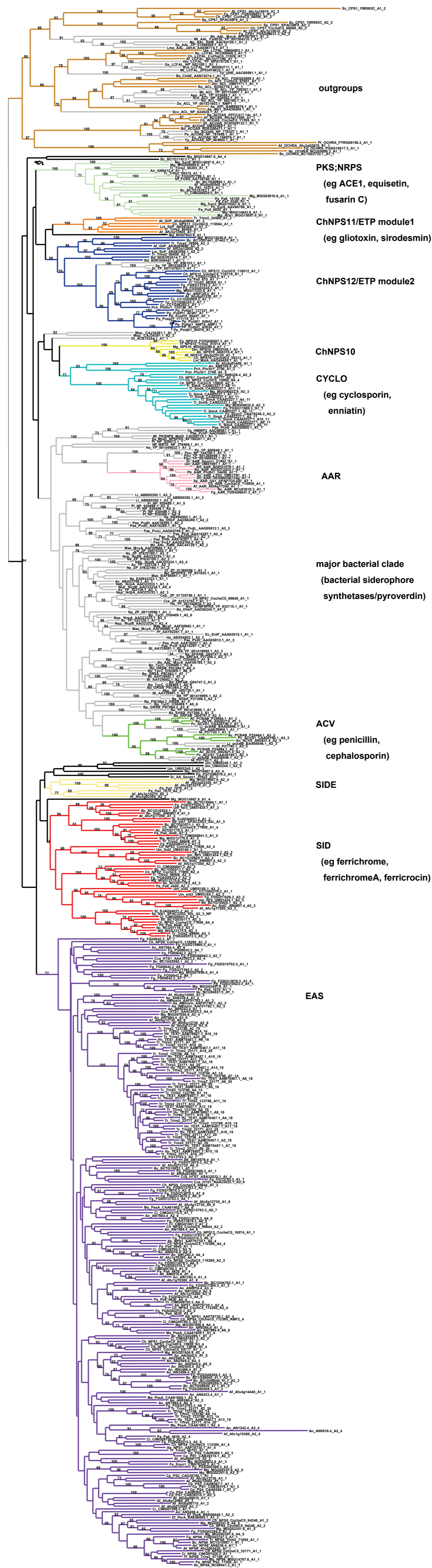

Supplement: Additional file 6 — Phylogenies, full dataset. A. NJ, B. ML (PhyML), and C. ML (RAxML) phylogenies of the full AMP dataset. See Additional file 15 for detailed description. [file 1471-2148-10-26-S6.PDF]

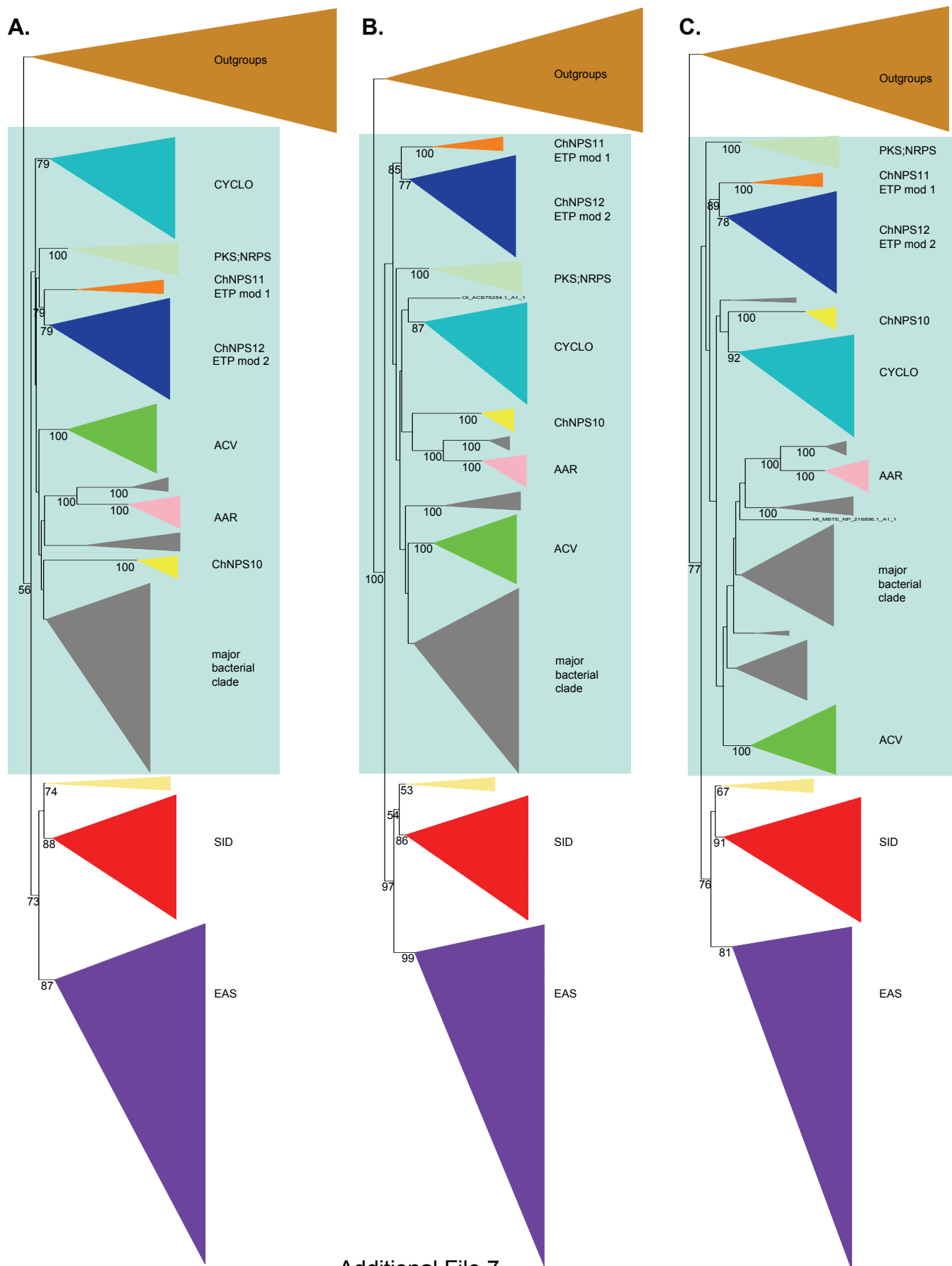

Supplement: Additional file 7 — Phylogenies, reduced dataset. Topologies of NJ, ML (PhyML), and ML (RAxML) phylogenetic analyses of the reduced NRPS AMP domain dataset containing selected representatives of each subfamily. See Additional file 15 for detailed description. [file 1471-2148-10-26-S7.PDF]
