# Supplementary material for: Phylogenomics reveals subfamilies of fungal nonribosomal peptide synthetases and their evolutionary relationships
Source: BMC Evol Biol. 2010 Jan 26;10:26. doi: 10.1186/1471-2148-10-26 (PMC2823734; doi:10.1186/1471-2148-10-26)
Supplement: Additional file 8 — Bacterial outgroup proteins. Bacterial proteins used as outgroups. [file 1471-2148-10-26-S8.PDF]

Additional File 8. Bacterial proteins used as outgroups

Species	NCBI Accession	Gene	Peptide Product	Reference
Anabaena variabilis (*Av*)	YP_322129.1			
Anabaena variabilis (*Av*)	ABA23700.1			
Bacillus amyloliquefaciens (*Ba*)	YP_001419995.1	SrfAA	Surfactin A	[1]
Bacillus subtilis (*Bs*)	AAD56240.1	DhbF	Bacillibactin	[2]
	P27206.3	SrfAA	Surfactin A	[3]
	AAN15214.1	DhbE	Bacillibactin	[4]
	Q04747.2	SrfAB	Surfactin B	[3]
Brevibacillus brevis (*Bb*)	P0C064.2	GrsB	Gramicidin B	[5]
	P0C062.1	GrsA	Gramicidin A	[6]
Brevibacillus parabrevis (*Bp*)	O30409.1	TycC	Tyrocidine C	[7]
Brevibacillus texasporus (*Bt*)	AAY29581.1	BtD	BT Peptide	[8]
	AAY29582.1	BtE	BT Peptide	[8]
Chlorobium ferrooxidans (*Cf*)	ZP_01386298.1			
Clostridium cellulolyticum (*Cce*)	ZP_01573792.1			
Crocosphaera watsonii (Cw)	ZP_00515352.1			
Cyanothece sp. CCY0110 (*Csp.*)	ZP_01728758.1			
Escherichia coli (*Eco*)	AAA92015.1	EntF	Enterobactin	[9]
Hahella chejuensis (*Hc*)	YP_436153.1			
Herpetosiphon aurantiacus (*Ha*)	YP_001544632.1			
	ABX04502.1			
Lysobacter lactamgenus (*Ll*)	BAA08846.1	pcbAB	Cephalosporin	[10]
	ABB80392.1	cpbI	(NRPS;PKS)	
Melittangium lichenicola (*Ml*)	CAD89775.1	MelD	Melithiazol (PKS;NRPS)	[11]
Microcystis aeruginosa (*Mae*)	AAF00960.1	McyA	Microcystin	[12]
	AAF00962.1	McyC	Microcystin	[12]
	BAF68991.1	psm3B	(NRPS;PKS)	[13]
Micromonospora sp. (*Msp.*)	CAJ34381.1	tioY	Thiocoraline	
Mycobacterium tuberculosis (*Mt*)	NP_216896.1	MBTE	MBTE siderophore	[14]
Myxococcus xanthus (*Mx*)	YP_631822.1		NRPS;PKS	[15]
	YP_632115.1	Ta1	NRPS;PKS	[15]
Nocardia lactamdurans (*Nl*)	P27743.1	pcbAB	Cephamycin	[16]
Nodularia spumigena (*Ns*)	EAW43322.1			
	ZP_01632190.1			
	ZP_01632190.1			
Nostoc punctiforme (*Np*)	ZP_00110590.1			
Nostoc sp. (*Nsp.*)	AAO23333.1	NcpA	4-Methylproline	[17]
	AAO23334.1	NcpB	4-Methylproline	[17]
Opitutus terrae (*Ot*)	ACB75254.1			
Photorhabdus luminescens subsp. *laumondii* (*Pl*)	NP_929573.1		NRPS;PKS	[18]
	NP_930489.1			[18]
Pseudomonas aeruginosa (*Pae*)	AAD55800.1	PchE	Pyochelin	[19]
	AAD55801.1	PchF	Pyochelin	[19]
	AAX16295.1	PvdD	Pyoverdine	[20]
	AAX16297.1	PvdI	Pyoverdine	[20]
	AAG05788.2	PvdJ	Pyoverdine	[21]
	AAG05812.1	PvdL	Pyoverdine	[21]
Pseudomonas entomophila (*Pe*)	YP_608846.1			[22]
Pseudomonas fluorescens (*Pf*)	AAY92261.1			[23]
Pseudomonas putida F1 (*Ppu*)	YP_001268464.1			[24]
	YP_001669542.1			[24]
	NP_744708.1			[24]

Salinispora arenicola CNS-205 (*Sar*)	YP_001535628.1			
Salinispora tropica (*St*)	YP_001157631.1			
Stigmatella aurantiaca (*Sau*)	AF188287.1	MtaA-MtaG	Myxothiazol	[25]
Streptomyces clavuligerus (*Scl*)	AAB39900.1	pcbAB	Penicillin	[26]
Yersinia pestis (*Yp*)	AAC69591.1	ybtE	Yersiniabactin	[27]
	AAC69587.1	HMWP2	Yersiniabactin	[27, 28]

Blank = unknown or not published

1. Chen XH, Koumoutsi A, Scholz R, Eisenreich A, Schneider K, Heinemeyer I, Morgenstern B, Voss B, Hess WR, Reva O *et al*: **Comparative analysis of the complete genome sequence of the plant growth-promoting bacterium *Bacillus amyloliquefaciens* FZB42**. *Nature Biotechnology* 2007, **25**(9):1007-1014.
2. May JJ, Wendrich TM, Marahiel MA: **The dhb operon of *Bacillus subtilis* encodes the biosynthetic template for the catecholic siderophore 2,3-dihydroxybenzoate-glycine-threonine trimeric ester bacillibactin**. *Journal of Biological Chemistry* 2001, **276**(10):7209-7217.
3. Fuma S, Fujishima Y, Corbell N, Dsouza C, Nakano MM, Zuber P, Yamane K: **Nucleotide-sequence of 5' portion of SrfA that contains the region required for competence establishment in *Bacillus subtilis***. *Nucleic Acids Research* 1993, **21**(1):93-97.
4. May JJ, Kessler N, Marahiel MA, Stubbs MT: **Crystal structure of DhbE, an archetype for aryl acid activating domains of modular nonribosomal peptide synthetases**. *Proceedings of the National Academy of Sciences of the United States of America* 2002, **99**(19):12120-12125.
5. Saito F, Hori K, Kanda M, Kurotsu T, Saito Y: **Entire nucleotide sequence for *Bacillus brevis nagano* Grs2 gene encoding Gramicidin-S synthetase 2 - a multifunctional peptide synthetase**. *Journal of Biochemistry* 1994, **116**(2):357-367.
6. Hori K, Yamamoto Y, Minetoki T, Kurotsu T, Kanda M, Miura S, Okamura K, Furuyama J, Saito Y: **Molecular cloning and nucleotide-sequence of the Gramicidin-S synthetase 1 gene**. *Journal of Biochemistry* 1989, **106**(4):639-645.
7. Mootz HD, Marahiel MA: **The tyrocidine biosynthesis operon of *Bacillus brevis*: Complete nucleotide sequence and biochemical characterization of functional internal adenylation domains**. *Journal of Bacteriology* 1997, **179**(21):6843-6850.
8. Wu XF, Ballard J, Jiang YW: **Structure and biosynthesis of the BT peptide antibiotic from *Brevibacillus texasporus***. *Applied and Environmental Microbiology* 2005, **71**(12):8519-8530.
9. Rusnak F, Sakaitani M, Drueckhammer D, Reichert J, Walsh CT: **Biosynthesis of the *Escherichia coli* siderophore enterobactin - sequence of the Entf gene, expression and purification of Entf, and analysis of covalent phosphopantetheine**. *Biochemistry* 1991, **30**(11):2916-2927.
10. Kimura H, Miyashita H, Sumino Y: **Organization and expression in *Pseudomonas putida* of the gene cluster involved in cephalosporin biosynthesis from *Lysobacter lactamgenus* YK90**. *Applied Microbiology and Biotechnology* 1996, **45**(4):490-501.
11. Weinig S, Hecht HJ, Mahmud T, Muller R: **Melithiazol biosynthesis: Further insights into myxobacterial PKS/NRPS systems and evidence for a new subclass of methyl transferases**. *Chemistry & Biology* 2003, **10**(10):939-952.
12. Tillett D, Dittmann E, Erhard M, von Dohren H, Borner T, Neilan BA: **Structural organization of microcystin biosynthesis in *Microcystis aeruginosa* PCC7806: an integrated peptide-polyketide synthetase system**. *Chemistry & Biology* 2000, **7**(10):753-764.
13. Nishizawa A, Bin Arshad A, Nishizawa T, Asayama M, Fujii K, Nakano T, Harada K, Shirai M: **Cloning and characterization of a new hetero-gene cluster of nonribosomal peptide synthetase and polyketide synthase from the cyanobacterium *Microcystis aeruginosa* K-139**. *Journal of General and Applied Microbiology* 2007, **53**(1):17-27.
14. Cole ST, Brosch R, Parkhill J, Garnier T, Churcher C, Harris D, Gordon SV, Eiglmeier K, Gas S, Barry CE *et al*: **Deciphering the biology of *Mycobacterium tuberculosis* from the complete genome sequence (vol 393, pg 537, 1998)**. *Nature* 1998, **396**(6707):190-198.

15. Goldman BS, Nierman WC, Kaiser D, Slater SC, Durkin AS, Eisen JA, Ronning CM, Barbazuk WB, Blanchard M, Field C *et al*: **Evolution of sensory complexity recorded in a myxobacterial genome**. *Proc Natl Acad Sci USA* 2006, **103**(41):15200-15205
16. Coque JJR, Martin JF, Calzada JG, Liras P: **The Cephamycin biosynthetic genes PcbAB, encoding a large multidomain peptide synthetase, and PcbC of *Nocardia lactamdurans* are clustered together in an organization different from the same genes in *Acremonium chrysogenum* and *Penicillium chrysogenum***. *Molecular Microbiology* 1991, **5**(5):1125-1133.
17. Luesch H, Hoffmann D, Hevel JM, Becker JE, Golakoti T, Moore RE: **Biosynthesis of 4-methylproline in cyanobacteria: Cloning of nosE and nosF genes and biochemical characterization of the encoded dehydrogenase and reductase activities**. *Journal of Organic Chemistry* 2003, **68**(1):83-91.
18. Duchaud E, Rusniok C, Frangeul L, Buchrieser C, Givaudan A, Taourit S, Bocs S, Boursaux-Eude C, Chandler M, Charles JF *et al*: **The genome sequence of the entomopathogenic bacterium *Photorhabdus luminescens***. *Nature Biotechnology* 2003, **21**(11):1307-1313.
19. Quadri LEN, Keating TA, Patel HM, Walsh CT: **Assembly of the *Pseudomonas aeruginosa* nonribosomal peptide siderophore pyochelin: In vitro reconstitution of aryl-4,2-bisthiazoline synthetase activity from PchD, PchE, and PchF**. *Biochemistry* 1999, **38**(45):14941-14954.
20. Smith EE, Sims EH, Spencer DH, Kaul R, Olson MV: **Evidence for diversifying selection at the pyoverdine locus of *Pseudomonas aeruginosa***. *Journal of Bacteriology* 2005, **187**(6):2138-2147.
21. Stover CK, Pham XQ, Erwin AL, Mizoguchi SD, Warrener P, Hickey MJ, Brinkman FSL, Hufnagle WO, Kowalik DJ, Lagrou M *et al*: **Complete genome sequence of *Pseudomonas aeruginosa* PAO1, an opportunistic pathogen**. *Nature* 2000, **406**(6799):959-964.
22. Vodovar N, Vallenet D, Cruveiller S, Rouy Z, Barbe V, Acosta C, Cattolico L, Jubin C, Lajus A, Segurens B *et al*: **Complete genome sequence of the entomopathogenic and metabolically versatile soil bacterium *Pseudomonas entomophila***. *Nat Biotechnol* 2006, **24**(6):673-679.
23. Paulsen IT, Press CM, Ravel J, Kobayashi DY, Myers GSA, Mavrodi DV, DeBoy RT, Seshadri R, Ren QH, Madupu R *et al*: **Complete genome sequence of the plant commensal *Pseudomonas fluorescens* Pf-5**. *Nature Biotechnology* 2005, **23**(7):873-878.
24. Nelson KE, Weinel C, Paulsen IT, Dodson RJ, Hilbert H, dos Santos V, Fouts DE, Gill SR, Pop M, Holmes M *et al*: **Complete genome sequence and comparative analysis of the metabolically versatile *Pseudomonas putida* KT2440**. *Environmental Microbiology* 2002, **4**(12):799-808.
25. Silakowski B, Schairer HU, Ehret H, Kunze B, Weinig S, Nordsiek G, Brandt P, Blocker H, Hofle G, Beyer S *et al*: **New lessons of combinatorial biosynthesis from myxobacteria - The myxothiazol biosynthetic gene cluster of *Stigmatella aurantiaca* DW4/3-1**. *Journal of Biological Chemistry* 1999, **274**(52):37391-37399.
26. Yu H, Serpe E, Romero J, Coque JJ, Maeda K, Oelgeschlager M, Hintermann G, Liras P, Martin JF, Demain AL *et al*: **Possible Involvement of the Lysine epsilon aminotransferase gene (Lat) in the expression of the genes encoding ACV Synthetase (PcbAB) and Isopenicillin-N synthase (PcbC) in *Streptomyces clavuligerus***. *Microbiology-Uk* 1994, **140**:3367-3377.
27. Gehring AM, DeMoll E, Fetherston JD, Mori I, Mayhew GF, Blattner FR, Walsh CT, Perry RD: **Iron acquisition in plague: modular logic in enzymatic biogenesis of yersiniabactin by *Yersinia pestis***. *Chemistry & Biology* 1998, **5**(10):573-586.
28. Gehring AM, Mori I, Perry RD, Walsh CT: **The nonribosomal peptide synthetase HMWP2 forms a thiazoline ring during biogenesis of Yersiniabactin, an iron-chelating virulence factor of *Yersinia pestis* (vol 37, pg 11637, 1998)**. *Biochemistry* 1998, **37**(48):17104-17104.
