# Supplementary material for: Phylogenomics reveals subfamilies of fungal nonribosomal peptide synthetases and their evolutionary relationships
Source: BMC Evol Biol. 2010 Jan 26;10:26. doi: 10.1186/1471-2148-10-26 (PMC2823734; doi:10.1186/1471-2148-10-26)
Supplement: Additional file 9 — Newick phylogenetic tree. Text file containing newick phylogenetic tree for opening in tree visualization programs such as Treeview [121]. See Additional file 15 for detailed description. [file 1471-2148-10-26-S9.PDF]

Additional File 9

The ultrametric species tree used in CAFE analyses was created using the PL method in r8s [1] and the phylogeny of the concatenated protein dataset of Fitzpatrick et al. [2]. We used 5 calibration points (Dikarya = 452 MYA, Basidiomycetes = 340 MYA, Ascomycetes = 400 MYA, Pezizomycetes = 215 MYA, and Sordariomycetes = 122 MYA) estimated by Taylor and Berbee [3] when fixing the 400 MYO fungal fossil *Paleopyenromycites devonicus* at the origin of the ascomycetes. As the root taxon was unconstrained, we estimated the date of the root taxon, *R. oryzae* based on previously published studies [4] to be 480MYA, less than Taylor and Berbee's estimate for the origin of fungi (495 MYA)[3]. Assigning the *Paleopyrenomycites* at the origins of ascomycota as opposed to the other suggested dates for this fossil (at the origins of Pyrenomycetes and Sordariomycetes respectively) gives time estimates for the origins of Glomeromycota best coinciding with the radiation of land plants [3].

Ultrametric Species Tree:

(Roryz:480,((Umayd:340,((Ccin:185,Pchry:185):102,Cneo:287):53)BA:112,(Spomb:400,((((Afum:72,Anid:72):91,Cimm:163):52,(Chet:183,(Bcin:153,((Trees:78,fgram:78):44,(Mgris:88,(Ncras:62,Pans:62):26):34)SO:31):30):32)EA:145,(Ylip:290,(((calb:71,ctrop:71):63,((dhans:94,Cguill:94):17,Clus:111):23):79,((klact:87,Agoss:87):26,(Sbay:20,(Smik:14,(Scer:10,Spar:10):4):6):93):101):77):70):40)AS:52)DK:28);

1. Sanderson MJ: **r8s: inferring absolute rates of molecular evolution and divergence times in the absence of a molecular clock**. *Bioinformatics* 2003, **19**(2):301-302.
2. Fitzpatrick DA, Logue, Mary E., Stajich, Jason E., and Butler, Geraldine: **A fungal phylogeny based on 42 complete genomes derived from supertree and combined gene analysis**. *BMC Evolutionary Biology* 2006, **6**:99.
3. Taylor JW, Berbee ML: **Dating divergences in the Fungal Tree of Life: review and new analyses**. *Mycologia* 2006, **98**(6):838-849.
4. Karlsson M, Stenlid J: **Comparative evolutionary histories of the fungal chitinase gene family reveal non-random size expansions and contractions due to adaptive natural selection**. *Evolutionary Bioinformatics* 2008:47-60.
