# Supplementary material for: Phylogenomics reveals subfamilies of fungal nonribosomal peptide synthetases and their evolutionary relationships
Source: BMC Evol Biol. 2010 Jan 26;10:26. doi: 10.1186/1471-2148-10-26 (PMC2823734; doi:10.1186/1471-2148-10-26)
Supplement: Additional file 10 — Known fungal NRPSs used for constructing the initial HMMER model. Known fungal NRPS AMP domains used for constructing the initial HMMER model. [file 1471-2148-10-26-S10.PDF]

Additional File 10. Known fungal NRPSs used for constructing the initial HMM model.

Species	NCBI Protein Accession #	Sequencing Center ID[a]	NRPS Name/Product	Reference[b]
Alternaria alternata	AAF01762.1		AMT/AM-toxin	[1]
Alternaria brassicae	AAP78735.1		NPS1	[2]
Acremonium chrysogenum	P25464.1		PCBAB/Cephalosporin	[3]
Aspergillus fumigatus	EAL88817.1	Afu6g09660	GliP/Gliotoxin	[4-6]
	EAL91592.1	Afu5g10120	NPS10	[5-7]
	EAL86624.1	Afu3g03420	NPS6/TAFC	[5-7]
Aspergillus nidulans	XP_660225.1	AN2621.4	ACVS/Penicillin	[8, 9]
Cochliobolus carbonum	AAA33023.1		HTS1/HC-toxin	[10]
Cochliobolus heterostrophus[c]	AAX09983.1		NPS1	[7]
	AAX09984.1		NPS2/ferricrocin	[7, 11]
	AAX09985.1		NPS3	[7]
	AAX09986.1		NPS4	[7]
	AAX09987.1		NPS5	[7]
	AAX09988.1		NPS6/coprogen	[7, 12]
	AAX09989.1		NPS7	[7]
	AAX09990.1		NPS8	[7]
	AAX09991.1		NPS9	[7]
	AAX09992.1		NPS10	[7]
	AAX09993.1		NPS11	[7]
	AAX09994.1		NPS12	[7]
Claviceps purpurea	CAB39315.1		PS1/D-lysergic acid	[13]
Epichloë festuca	BAE06845.1		PerA/Peramine	[14]
Fusarium equiseti	CAA79245.2		Esyn1/Enniatin	[15]
Fusarium graminearum	XP_383923.1	FG03747.1	NPS6/coprogen	[7, 16]
	XP_386683.1	FGSG_06507.3	NPS10	[7, 16]
	XP_383923.1	FG03747.1	NPS6/coprogen	[7, 16]
Fusarium heterosporum	AAV66106.1		EqiS/Equisetin	[17]
Gibberella fujikuroi	AAT28740.1		FUSS/Fusarin C	[18]
Hypocrea virens	AAM78457.1		TEX1/peptaibol	[19]
Leptosphaeria maculans	AAO49458.1		MAA	[20]
	AAS92545.1		SirP/sirodesmin PL	[21]
Metarhizium anisopliae	CAA61605.1		PesA	[22]
Magnaporthe oryzae	CAG28798.1	MGG_15097.6	Ace1	[23]
	XP_360747.1	MGG_03290.6	NPS10	[7, 24]
	XP_364124.2	MGG_14767.6	NPS6/coprogen	[7, 24]
	CAG28798.1	MGG_12447.6	Syn2	[23]
	CAH59193.1	MGG_12447.6	Syn8	[23]
Neurospora crassa	XP_963411.2	NCU_08441.3	NPS6/coprogen	[7, 25]
Penicillium chrysogenum	CAA38195.1		ACVS1/Penicillin	[26]
	CAD28788.1		PS2/ergotamine	[27]
	CAI59267.1		PS3	[27]
	CAI59268.1		PS4/ergocryptine	[27]
Schizosaccharomyces pombe	CAB88271.1		Lys1/ a-aminoadipate reductase	8, 29]
Tolypocladium inflatum	CAA82227.1		SimA/Cyclosporin	[30]
Ustilago maydis	XP_759255.1	UM03108.1	NPS10	[7, 31]
	AAB93493.1	UM05165.1	sid2/ferrichrome	[31, 32]
	XP_757581.1	UM01434.1	fer3/ferrichrome A	[31, 33]

[a] Blank = not applicable, [b] Blank = unpublished, [c] From *C. heterostrophus* strain C4

References

1. Johnson RD, Johnson L, Itoh Y, Kodama M, Otani H, Kahmoto K: **Cloning and characterization of a cyclic peptide synthetase gene from *Alternaria alternata* apple pathotype whose product is involved in AM-toxin synthesis and pathogenicity**. *Molecular Plant-Microbe Interactions* 2000, **13**(7):742-753.
2. Guillemette T, Sellam A, Simoneau P: **Analysis of a nonribosomal peptide synthetase gene from *Alternaria brassicae* and flanking genomic sequences**. *Current Genetics* 2004, **45**(4):214-224.
3. Gutierrez S, Diez B, Montenegro E, Martin JF: **Characterization of the *Cephalosporium acremonium pcbAB* gene encoding alpha-aminoadipyl-cysteinyl-valine synthetase, a large multidomain peptide synthetase - linkage to the pcbc gene as a cluster of early Cephalosporin biosynthetic genes and evidence of multiple functional domains**. *Journal of Bacteriology* 1991, **173**(7):2354-2365.
4. Cramer RA, Gamcsik MP, Brooking RM, Najvar LK, Kirkpatrick WR, Patterson TF, Balibar CJ, Graybill JR, Perfect JR, Abraham SN *et al*: **Disruption of a nonribosomal peptide synthetase in *Aspergillus fumigatus* eliminates gliotoxin production**. *Eukaryotic Cell* 2006, **5**(6):972-980.
5. Cramer RA, Stajich, J.E., Yvonne Yamanaka, Dietrich, F.S., Steinbach, William, J.S., and Perfect, J.R.: **Phylogenomic analysis of non-ribosomal peptide synthetases in the genus *Aspergillus***. *Gene* 2006, **383**(15):24-32.
6. Nierman WC, Pain A, Anderson MJ, Wortman JR, Kim HS, Arroyo J, Berriman M, Abe K, Archer DB, Bermejo C *et al*: **Genomic sequence of the pathogenic and allergenic filamentous fungus *Aspergillus fumigatus* (vol 438, pg 1151, 2005)**. *Nature* 2006, **439**(7075):502-502.
7. Lee BN, Kroken S, Chou DYT, Robbertse B, Yoder OC, Turgeon BG: **Functional analysis of all nonribosomal peptide synthetases in *Cochliobolus heterostrophus* reveals a factor, NPS6, involved in virulence and resistance to oxidative stress**. *Eukaryotic Cell* 2005, **4**(3):545-555.
8. Maccabe AP, Vanliempt H, Palissa H, Unkles SE, Riach MBR, Pfeifer E, Vondohren H, Kinghorn JR: **Delta-(L-alpha-aminoadipyl)-l-cysteinyl-d-valine synthetase from *Aspergillus nidulans* - Molecular characterization of the acvA gene encoding the 1st enzyme of the penicillin biosynthetic pathway**. *Journal of Biological Chemistry* 1991, **266**(19):12646-12654.
9. Galagan JE, Calvo SE, Cuomo C, Ma LJ, Wortman JR, Batzoglou S, Lee SI, Basturkmen M, Spevak CC, Clutterbuck J *et al*: **Sequencing of *Aspergillus nidulans* and comparative analysis with *A. fumigatus* and *A. oryzae***. *Nature* 2005, **438**(7071):1105-1115.
10. Scottcraig JS, Panaccione DG, Pocard JA, Walton JD: **the cyclic peptide synthetase catalyzing HC-toxin production in the filamentous fungus *Cochliobolus carbonum* is encoded by a 15.7-kilobase open reading frame**. *Journal of Biological Chemistry* 1992, **267**(36):26044-26049.
11. Oide S, Krasnoff SB, Gibson DM, Turgeon BG: **Intracellular siderophores are essential for ascomycete sexual development in heterothallic *Cochliobolus heterostrophus* and homothallic *Gibberella zeae***. *Eukaryotic Cell* 2007, **6**(8):1339-1353.
12. Oide S, Moeder W, Krasnoff S, Gibson D, Haas H, Yoshioka K, Turgeon BG: **NPS6, encoding a nonribosomal peptide synthetase involved in siderophore-mediated iron metabolism, is a conserved virulence determinant of plant pathogenic ascomycetes**. *Plant Cell* 2006, **18**(10):2836-2853.
13. Tudzynski P, Hoelter K, Correia T, Arntz C, Grammel N, Keller U: **Evidence for an ergot alkaloid gene cluster in *Claviceps purpurea***. *Molecular and General Genetics* 1999, **261**(1):133-141.
14. Tanaka A, Tapper BA, Popay A, Parker EJ, Scott B: **A symbiosis expressed non-ribosomal peptide synthetase from a mutualistic fungal endophyte of perennial ryegrass confers protection to the symbiotum from insect herbivory**. *Molecular Microbiology* 2005, **57**(4):1036-1050.
15. Haese A, Pieper R, Vonostrowski T, Zocher R: **Bacterial expression of catalytically active fragments of the multifunctional enzyme enniatin synthetase**. *Journal of Molecular Biology* 1994, **243**(1):116-122.
16. Cuomo CA, Gueldener U, Xu JR, Trail F, Turgeon BG, Di Pietro A, Walton JD, Ma LJ, Baker SE, Rep M *et al*: **The *Fusarium graminearum* genome reveals a link between localized polymorphism and pathogen specialization**. *Science* 2007, **317**(5843):1400-1402.

17. Sims JW, Fillmore JP, Warner DD, Schmidt EW: **Equisetin biosynthesis in *Fusarium heterosporum***. *Chemical Communications* 2005(2):186-188.
18. Song ZS, Cox RJ, Lazarus CM, Simpson TJ: **Fusarin C biosynthesis in *Fusarium moniliforme* and *Fusarium venenatum***. *Chembiochem* 2004, **5**(9):1196-1203.
19. Wiest A, Grzegorski D, Xu BW, Goulard C, Rebuffat S, Ebbole DJ, Bodo B, Kenerley C: **Identification of peptaibols from *Trichoderma virens* and cloning of a peptaibol synthetase**. *Journal of Biological Chemistry* 2002, **277**(23):20862-20868.
20. Idnurm A, Taylor JL, Pedras MSC, Howlett BJ: **Small scale functional genomics of the blackleg fungus, *Leptosphaeria maculans*: analysis of a 38 kb region**. *Australasian Plant Pathology* 2003, **32**(4):511-519.
21. Gardiner DM, Cozijnsen AJ, Wilson LM, Pedras MSC, Howlett BJ: **The sirodesmin biosynthetic gene cluster of the plant pathogenic fungus *Leptosphaeria maculans***. *Molecular Microbiology* 2004, **53**(5):1307-1318.
22. Bailey AM, Kershaw MJ, Hunt BA, Paterson IC, Charnley AK, Reynolds SE, Clarkson JM: **Cloning and sequence analysis of an intron-containing domain from a peptide synthetase-encoding gene of the entomopathogenic fungus *Metarhizium anisopliae***. *Gene* 1996, **173**(2):195-197.
23. Bohnert HU, Fudal I, Dioh W, Tharreau D, Notteghem JL, Lebrun MH: **A putative polyketide synthase peptide synthetase from *Magnaporthe grisea* signals pathogen attack to resistant rice**. *Plant Cell* 2004, **16**(9):2499-2513.
24. Dean RA, Talbot NJ, Ebbole DJ, Farman ML, Mitchell TK, Orbach MJ, Thon M, Kulkarni R, Jin-Rong X, Huaqin P *et al*: **The genome sequence of the rice blast fungus *Magnaporthe grisea***. *Nature* 2005, **434**(7036):980(987).
25. Galagan JE, Calvo SE, Borkovich KA, Selker EU, Read ND, Jaffe D, FitzHugh W, Ma LJ, Smirnov S, Purcell S *et al*: **The genome sequence of the filamentous fungus *Neurospora crassa***. *Nature* 2003, **422**(6934):859-868.
26. Smith DJ, Earl AJ, Turner G: **The multifunctional peptide synthetase performing the 1st step of Penicillin biosynthesis in *Penicillium chrysogenum* is a 421-073 Dalton protein similar to *Bacillus brevis* peptide antibiotic synthetases**. *Embo Journal* 1990, **9**(9):2743-2750.
27. Haarmann T, Machado C, Lubbe Y, Correia T, Schardl CL, Panaccione DG, Tudzynski P: **The ergot alkaloid gene cluster in *Claviceps purpurea*: Extension of the cluster sequence and intra species evolution**. *Phytochemistry* 2005, **66**(11):1312-1320.
28. Guo S, Bhattacharjee JK: **Posttranslational activation, site-directed mutation and phylogenetic analyses of the lysine biosynthesis enzymes alpha-aminoadipate reductase Lys1p (AAR) and the phosphopantetheinyl transferase Lys7p (PPTase) from *Schizosaccharomyces pombe***. *Yeast* 2004, **21**(15):1279-1288.
29. Wood V, Gwilliam R, Rajandream MA, Lyne M, Lyne R, Stewart A, Sgouros J, Peat N, Hayles J, Baker S *et al*: **The genome sequence of *Schizosaccharomyces pombe***. *Nature* 2002, **415**(6874):871-880.
30. Weber G, Schorgendorfer K, Schneiderscherzer E, Leitner E: **The peptide synthetase catalyzing Cyclosporine production in *Tolypocladium niveum* is encoded by a giant 45.8-kilobase open reading frame**. *Current Genetics* 1994, **26**(2):120-125.
31. Kamper J, Kahmann R, Bolker M, Ma LJ, Brefort T, Saville BJ, Banuett F, Kronstad JW, Gold SE, Muller O *et al*: **Insights from the genome of the biotrophic fungal plant pathogen *Ustilago maydis***. *Nature* 2006, **444**(7115):97-101.
32. Yuan WM, Gentil, Guillaume D., Budde, Allen D., and Leong, Sally A.: **Characterization of the *Ustilago maydis sid2* Gene, encoding a multidomain peptide synthetase in the ferrichrome biosynthetic gene cluster**. *Journal of Bacteriology* 2001, **183**(13):4040-4051.
33. Eichhorn H, Lessing F, Winterberg B, Schirawski J, Kamper J, Muller P, Kahmann R: **A ferroxidation/permeation iron uptake system is required for virulence in *Ustilago maydis***. *Plant Cell* 2006, **18**(11):3332-3345.
