# Supplementary material for: Phylogenomics reveals subfamilies of fungal nonribosomal peptide synthetases and their evolutionary relationships
Source: BMC Evol Biol. 2010 Jan 26;10:26. doi: 10.1186/1471-2148-10-26 (PMC2823734; doi:10.1186/1471-2148-10-26)
Supplement: Additional file 12 — Fungal protein datasets used in phylogenomic analyses. Fungal protein (AMP domain) datasets used in phylogenomic analyses. [file 1471-2148-10-26-S12.PDF]

Additional File 12. Fungal Protein Datasets used in phylogenomic analyses

Classification/Species	Lifestyle	URL	Reference [a]
**Chytridiomycota**			
Batrachochytrium dendrobatidis (JEL423)	animal pathogen	http://www.broad.mit.edu/annotation/genome/batrachochytrium_dendrobatidis/	
**Zygomycota**			
Rhizopus oryzae (RA99-880)	saprobe	http://www.broad.mit.edu/annotation/genome/rhizopus_oryzae/MultiHome.html	
Phycomyces blakesleeanus (NRRL1555)	saprobe	http://genome.jgi-psf.org/Phybl1/Phybl1.home.html	
**Microsporidia**			
Enchephalitozoon cuniculi (GB-M1)	animal pathogen	http://www.genoscope.cns.fr/spip/Encephalitozoon-cuniculi-whole.html	[1]
**Schizosaccharomycota**			
Schizosaccharomyces pombe (972h)	saprobe	http://www.broad.mit.edu/annotation/genome/schizosaccharomyces_group/MultiHome.html	[2]
Schizosaccharomyces japonicus (yFS275)	saprobe	http://www.broad.mit.edu/annotation/genome/schizosaccharomyces_group/MultiHome.html	
**Hemiascomycota**			
Ashbya gossypii (ATCC 10895)	plant pathogen	Ashbya Genome Database: http://agd.vital-it.ch/index.html	[3]
Candida albicans (WO1)	animal pathogen	http://www.broad.mit.edu/annotation/genome/candida_group/MultiHome.html	[4]
Candida glabrata (CBS138)	animal pathogen	http://www.genolevures.org/cagl.html#	[5]
Candida guilliermondii (ATCC6260)	animal pathogen	http://www.broad.mit.edu/annotation/genome/candida_group/MultiHome.html	
Candida lusitaniae (ATCC42720)	animal pathogen	http://www.broad.mit.edu/annotation/genome/candida_group/MultiHome.html	
Candida tropicalis (CBS94)	animal pathogen	http://www.broad.mit.edu/annotation/genome/candida_group/MultiHome.html	[6]
Saccharomyces cerevisiae (S288C)	saprobe	http://www.yeastgenome.org/	[7]
Saccharomyces paradoxicus (NRRLY-17217)	saprobe	Broad Institute, GenBank Accession AABZ00000000	[8]
Saccharomyces bayanus (MCYC623)	saprobe	Broad Institute, GenBank Accession AACA00000000	[8]
Saccharomyces mikatae (IFO1815)	saprobe	Broad Instiute, GenBank Accession AABZ00000000	[8]
Debaryomyces hansenii (CBS767)	saprobe	http://www.genolevures.org/deha.html#	[5]
Kluyveromyces lactis var. *lactis* (CLIB210)	saprobe	http://www.genolevures.org/klla.html#	[5]
Yarrowia lipolytica (CLIB99)	saprobe	http://www.genolevures.org/yali.html#	[5]

**Euascomycota**			
Aspergillus nidulans (FGSC A4)	saprobe	http://www.broad.mit.edu/annotation/genome/aspergillus_group/MultiHome.html	[9]
Aspergillus fumigatus (*Af293*)	animal pathogen	CADRE: http://www.cadre-genomes.org.uk/aspergillus_links.html	[10]
Magnaporthe oryzae (*70-15*)	plant pathogen	http://www.broad.mit.edu/annotation/genome/magnaporthe_grisea/MultiHome.html	[11]
Fusarium graminearum (*PH-1*)	plant pathogen	http://www.broad.mit.edu/annotation/genome/fusarium_group/MultiHome.html	[12]
Botrytis cinerea (B05.10)	plant pathogen	http://www.broad.mit.edu/annotation/genome/botrytis_cinerea/	
Coccidioides immitis (RS)	animal pathogen	http://www.broad.mit.edu/annotation/genome/coccidioides_group/MultiHome.html	
Cochliobolus heterostrophus (C5)	plant pathogen	http://genome.jgi-psf.org/CocheC5_1/CocheC5_1.home.html	
Neurospora crassa (OR74A)	saprobe	http://www.broad.mit.edu/annotation/genome/neurospora/	[13]
Podospora anserina (DSM 980)	saprobe	http://podospora.igmors.u-psud.fr/	[14]
Trichoderma reesii (QM6a)	Mycoparasite	http://genome.jgi-psf.org/Trire2/Trire2.home.html	[15]
**Basidiomycota:**			
Coprinopsis cinerea (Okayama 7#130)	saprobe	http://www.broad.mit.edu/annotation/genome/coprinus_cinereus/MultiHome.html	
Cryptococcus neoforman var. grubii (serotype A – H99)	animal pathogen	http://www.broad.mit.edu/annotation/genome/cryptococcus_neoformans/MultiHome.html	[16]
Picia stipitis (NRRL Y-11545)	saprobe	http://genome.jgi-psf.org/Picst3/Picst3.home.html	[17]
Puccinia graminis *(*CRL 75-36-700-3)	plant pathogen	http://www.broad.mit.edu/annotation/genome/puccinia_graminis/	
Postia placenta (Mad-698-R)	saprobe	http://genome.jgi-psf.org/Pospl1/Pospl1.home.html	
Phanaerochaete chrysosporium (RP78)	saprobe	http://genome.jgi-psf.org/Phchr1/Phchr1.home.html	[18]
Laccaria bicolor (S238N-H82)	saprobe	http://genome.jgi-psf.org/Lacbi1/Lacbi1.home.html	[19]
Sporobolomyces roseus	saprobe	http://genome.jgi-psf.org/Sporo1/Sporo1.home.html	
Ustilago maydis (521)	plant pathogen	http://www.broad.mit.edu/annotation/genome/ustilago_maydis/	[20]

[a] Blank = unpublished

Reference

1. Katinka MD, Duprat S, Cornillot E, Metenier G, Thomarat F, Prensier G, Barbe V, Peyretaillade E, Brottier P, Wincker P *et al*: **Genome sequence and gene compaction of the eukaryote parasite *Encephalitozoon cuniculi***. *Nature* 2001, **414**(6862):450-453.
2. Wood V, Gwilliam R, Rajandream MA, Lyne M, Lyne R, Stewart A, Sgouros J, Peat N, Hayles J, Baker S *et al*: **The genome sequence of *Schizosaccharomyces pombe* (vol 415, pg 871, 2002)**. *Nature* 2003, **421**(6918):94-94.
3. Dietrich FS, Voegeli S, Brachat S, Lerch A, Gates K, Steiner S, Mohr C, Pohlmann R, Luedi P, Choi SD *et al*: **The *Ashbya gossypii* genome as a tool for mapping the ancient Saccharomyces cerevisiae genome**. *Science* 2004, **304**(5668):304-307.

4. Jones T, Federspiel NA, Chibana H, Dungan J, Kalman S, Magee BB, Newport G, Thorstenson YR, Agabian N, Magee PT *et al*: **The diploid genome sequence of *Candida albicans*.** *Proceedings of the National Academy of Sciences of the United States of America* 2004, **101**(19):7329-7334.
5. Dujon B, Sherman D, Fischer G, Durrens P, Casaregola S, Lafontaine I, de Montigny J, Marck C, Neuveglise C, Talla E *et al*: **Genome evolution in yeasts**. *Nature* 2004, **430**(6995):35-44.
6. Souciet JL, Aigle M, Artiguenave F, Blandin G, Bolotin-Fukuhara M, Bon E, Brottier P, Casaregola S, de Montigny J, Dujon B *et al*: **Genomic exploration of the hemiascomycetous yeasts: 1. A set of yeast species for molecular evolution studies**. *Febs Letters* 2000, **487**(1):3-12.
7. Goffeau A, Barrell BG, Bussey H, Davis RW, Dujon B, Feldmann H, Galibert F, Hoheisel JD, Jacq C, Johnston M *et al*: **Life with 6000 genes**. *Science* 1996, **274**(5287):546-547.
8. Kellis M, Patterson N, Endrizzi M, Birren B, Lander ES: **Sequencing and comparison of yeast species to identify genes and regulatory elements**. *Nature* 2003, **423**(6937):241-254.
9. Galagan JE, Calvo SE, Cuomo C, Ma LJ, Wortman JR, Batzoglou S, Lee SI, Basturkmen M, Spevak CC, Clutterbuck J *et al*: **Sequencing of *Aspergillus nidulans* and comparative analysis with *A. fumigatus* and *A. oryzae*.** *Nature* 2005, **438**(7071):1105-1115.
10. Nierman WC, Pain A, Anderson MJ, Wortman JR, Kim HS, Arroyo J, Berriman M, Abe K, Archer DB, Bermejo C *et al*: **Genomic sequence of the pathogenic and allergenic filamentous fungus *Aspergillus fumigatus* (vol 438, pg 1151, 2005)**. *Nature* 2006, **439**(7075):502-502.
11. Dean RA, Talbot NJ, Ebbole DJ, Farman ML, Mitchell TK, Orbach MJ, Thon M, Kulkarni R, Jin-Rong X, Huaqin P *et al*: **The genome sequence of the rice blast fungus Magnaporthe grisea**. *Nature* 2005, **434**(7036):980(987).
12. Cuomo CA, Gueldener U, Xu JR, Trail F, Turgeon BG, Di Pietro A, Walton JD, Ma LJ, Baker SE, Rep M *et al*: **The *Fusarium graminearum* genome reveals a link between localized polymorphism and pathogen specialization**. *Science* 2007, **317**(5843):1400-1402.
13. Galagan JE, Calvo SE, Borkovich KA, Selker EU, Read ND, Jaffe D, FitzHugh W, Ma LJ, Smirnov S, Purcell S *et al*: **The genome sequence of the filamentous fungus *Neurospora crassa*.** *Nature* 2003, **422**(6934):859-868.
14. Paoletti M, Saupe SJ: **The genome sequence of *Podospora anserina*, a classic model fungus**. *Genome Biology* 2008, **9**(5).
15. Martinez D, Berka RM, Henrissat B, Saloheimo M, Arvas M, Baker SE, Chapman J, Chertkov O, Coutinho PM, Cullen D *et al*: **Genome sequencing and analysis of the biomass-degrading fungus *Trichoderma reesei* (syn. *Hypocrea jecorina*) (vol 26, pg 553, 2008)**. *Nature Biotechnology* 2008, **26**(10):1193-1193.
16. Loftus BJ, Fung E, Roncaglia P, Rowley D, Amedeo P, Bruno D, Vamathevan J, Miranda M, Anderson IJ, Fraser JA *et al*: **The genome of the basidiomycetous yeast and human pathogen *Cryptococcus neoformans***. *Science* 2005, **307**(5713):1321-1324.
17. Jeffries TW, Grigoriev IV, Grimwood J, Laplaza JM, Aerts A, Salamov A, Schmutz J, Lindquist E, Dehal P, Shapiro H *et al*: **Genome sequence of the lignocellulose-bioconverting and xylose-fermenting yeast *Pichia stipitis***. *Nature Biotechnology* 2007, **25**(3):319-326.
18. Martinez D, Larrondo LF, Putnam N, Gelpke MDS, Huang K, Chapman J, Helfenbein KG, Ramaiya P, Detter JC, Larimer F *et al*: **Genome sequence of the lignocellulose degrading fungus *Phanerochaete chrysosporium* strain RP78 (vol 22, pg 695, 2004)**. *Nature Biotechnology* 2004, **22**(7):899-899.
19. Martin F, Aerts A, Ahren D, Brun A, Danchin EGJ, Duchaussoy F, Gibon J, Kohler A, Lindquist E, Pereda V *et al*: **The genome of *Laccaria bicolor* provides insights into mycorrhizal symbiosis**. *Nature* 2008, **452**(7183):88-U87.
20. Kamper J, Kahmann R, Bolker M, Ma LJ, Brefort T, Saville BJ, Banuett F, Kronstad JW, Gold SE, Muller O *et al*: **Insights from the genome of the biotrophic fungal plant pathogen *Ustilago maydis***. *Nature* 2006, **444**(7115):97-101.
