# Supplementary material for: Phylogenomics reveals subfamilies of fungal nonribosomal peptide synthetases and their evolutionary relationships
Source: BMC Evol Biol. 2010 Jan 26;10:26. doi: 10.1186/1471-2148-10-26 (PMC2823734; doi:10.1186/1471-2148-10-26)
Supplement: Additional file 15 — Additional file figure legends and notes. Selected additional file figure legends and notes. [file 1471-2148-10-26-S15.PDF]

Additional file 15. Additional file legends and notes

Additional file 1. Diagram of *Cochliobolus heterostrophus* NRPSs and their domain structure.

Included are 12 NRPSs, one NRPS;PKS hybrid (NPS7/PKS24), one AAR, and one pseudogene (NPS13). Annotation of domains shows that, with the exception of the duplicated copy of ChNPS12, each has an unique domain architecture. Domain abbreviations: Adenylation (A), Thiolation (T), Condensation (C), Epimerization (E), Methylation (M), Thioester reductase (R), Beta-ketosynthase (KS), Acyl transferase (AT), Dehydratase (DH), Ketoreductase (KR), and Ferric transmembrane reductase (FeR). Length of each gene in (bp) is shown to the right.

Additional file 4. HMMER fungal-specific models for A, T, and C domains.

Additional file 6. Phylogenies resulting from analyses of the full A domain dataset.

A. NJ tree using a ML distance matrix created using the WAG plus gamma model,

B. ML tree (PhyML) using the WAG plus gamma model, and

C. ML tree (RAxML) using the RTREVF plus gamma model.

Bootstrap support greater than 50% is shown under branches, where possible. Branches of monophyletic group defining subfamilies are color coded: brown: adenylating enzyme outgroups; light green: fungal PKS;NRPS hybrid synthetases (PKS:NRPS); dark orange: ChNPS11/ETP module 1 synthetases (ChNPS11/ETP mod1); dark blue: ChNPS12/ETP module 2 synthetases (ChNPS12/ETP mod2); yellow: ChNPS10-like synthetases (ChNPS10); light blue: Cyclosporin synthetases (CYCLO); pink: α-aminoadipate reductases (AAR); dark green: ACV synthetases (ACV); red: siderophore synthetases (SID); purple: Euascomycete clade synthetases (EAS). The majority of bacterial sequences (dark gray) group together and contain some fungal A domains (ACV synthetases and the NPS;PKS hybrid (ChNPS7;PKS24). The

remaining bacterial A domains group with the mono/bi-modular AAR and ChNPS12/ETP mod 2 subfamilies.

Additional file 7. Tree topologies from phylogenetic analyses of reduced dataset including representative A domains from each of the major fungal NRPS subfamilies.

A). NJ tree using a ML distance matrix created using the WAG plus gamma model, B). ML tree (PhyML) using the WAG plus gamma model, and C). ML tree (RAxML) using the RTREVF plus gamma model. Bootstrap support greater than 50% is shown under branches. Color coding as in Additional file 6 and Figure 1. Topologies for the reduced dataset show stronger bootstrap support (>70%) for grouping the multimodular and exclusively fungal SID and EAS clades together, than do the trees resulting from analysis of the full A domain dataset.

Additional file 9. Ultrametric species tree used for CAFÉ analyses. Tree was created with the PL method in r8s (Sanderson 2003) using the phylogeny of the concatenated protein dataset of Fitzpatrick et al. (Fitzpatrick 2006). We used 5 calibration points (Dikarya = 452 MYA, Basidiomycetes = 340 MYA, Ascomycetes 400 MYA, Pezizomycetes = 215 MYA, and Sordariomycetes = 122 MYA) estimated previously by Taylor and Berbee (Taylor and Berbee 2006) when fixing the 400 MYO fungal fossil *Paleopyenromycites devonicus* at the origin of the ascomycetes. The root taxon *R. oryzae* was constrained to be less than the origin of the Fungi (495 MYA) estimated in this study (Taylor and Berbee 2006). Assigning the *Paleopyrenomycites* at the origins of ascomycota as opposed to the other suggested dates for this fossil (at the origins of Pyrenomycetes and Sordariomycetes respectively) gives time estimates for the origins of Glomeromycota best coinciding with the radiation of land plants (Taylor and Berbee 2006).

Fitzpatrick, D. A., Logue, Mary E., Stajich, Jason E., and Butler, Geraldine (2006). "A fungal phylogeny based on 42 complete genomes derived from supertree and combined gene analysis." BMC Evolutionary Biology **6**: 99.

Sanderson, M. J. (2003). "r8s: inferring absolute rates of molecular evolution and divergence times in the absence of a molecular clock." Bioinformatics **19**(2): 301-302.

Taylor, J. W. and M. L. Berbee (2006). "Dating divergences in the Fungal Tree of Life: review and new analyses." Mycologia **98**(6): 838-849.

Additional file 13. Alignment of 558 fungal and bacterial AMP domains used in the complete AMP domain dataset phylogenetic analysis. Protein sequences of AMP domains were aligned with MUSCLE, manually adjusted, and masked to remove columns in the alignment with greater than 30% gaps.

Additional file 14. Alignment of reduced dataset of fungal and bacterial AMP domains containing selected representatives of each major fungal subfamily and bacterial clades. Protein sequences of AMP domains were aligned with MUSCLE and masked to remove columns with 50% of gaps.
